# Anaplerotic Pathways in *Halomonas elongata*: The Role of the Sodium Gradient

**DOI:** 10.3389/fmicb.2020.561800

**Published:** 2020-09-25

**Authors:** Karina Hobmeier, Marie C. Goëss, Christiana Sehr, Sebastian Schwaminger, Sonja Berensmeier, Andreas Kremling, Hans Jörg Kunte, Katharina Pflüger-Grau, Alberto Marin-Sanguino

**Affiliations:** ^1^Professorship for Systems Biotechnology, Technical University of Munich, Munich, Germany; ^2^Bioseparation Engineering Group, Department of Mechanical Engineering, Technical University of Munich, Munich, Germany; ^3^Division of Biodeterioration and Reference Organisms, Bundesanstalt Für Materialforschung und -Prüfung (BAM), Berlin, Germany

**Keywords:** thermodynamics-based metabolic flux analysis, halophilic bacteria, metabolic modeling, design principles, biochemistry and metabolism, *Halomonas elongata*

## Abstract

Salt tolerance in the γ-proteobacterium *Halomonas elongata* is linked to its ability to produce the compatible solute ectoine. The metabolism of ectoine production is of great interest since it can shed light on the biochemical basis of halotolerance as well as pave the way for the improvement of the biotechnological production of such compatible solute. Ectoine belongs to the biosynthetic family of aspartate-derived amino-acids. Aspartate is formed from oxaloacetate, thereby connecting ectoine production to the anaplerotic reactions that refill carbon into the tricarboxylic acid cycle (TCA cycle). This places a high demand on these reactions and creates the need to regulate them not only in response to growth but also in response to extracellular salt concentration. In this work, we combine modeling and experiments to analyze how these different needs shape the anaplerotic reactions in *H. elongata*. First, the stoichiometric and thermodynamic factors that condition the flux distributions are analyzed, then the optimal patterns of operation for oxaloacetate production are calculated. Finally, the phenotype of two deletion mutants lacking potentially relevant anaplerotic enzymes: phosphoenolpyruvate carboxylase (Ppc) and oxaloacetate decarboxylase (Oad) are experimentally characterized. The results show that the anaplerotic reactions in *H. elongata* are indeed subject to evolutionary pressures that differ from those faced by other gram-negative bacteria. Ectoine producing halophiles must meet a higher metabolic demand for oxaloacetate and the reliance of many marine bacteria on the Entner-Doudoroff pathway compromises the anaplerotic efficiency of Ppc, which is usually one of the main enzymes fulfilling this role. The anaplerotic flux in *H. elongata* is contributed not only by Ppc but also by Oad, an enzyme that has not yet been shown to play this role *in vivo*. Ppc is necessary for *H. elongata* to grow normally at low salt concentrations but it is not required to achieve near maximal growth rates as long as there is a steep sodium gradient. On the other hand, the lack of Oad presents serious difficulties to grow at high salt concentrations. This points to a shared role of these two enzymes in guaranteeing the supply of oxaloacetate for biosynthetic reactions.

## 1. Introduction

The halophilic γ-proteobacterium *Halomonas elongata* DSM 2581^*T*^ has a broad salt tolerance and can even grow in salt saturated brines (>30% NaCl) (Vreeland et al., 1980) thanks to the accumulation of the compatible solute ectoine, which can reach molar concentrations in the cytoplasm without disrupting cellular processes (Galinski, 1995). The synthetic pathway for ectoine is well-known (Ono et al., 1999). Ectoine is synthesized from aspartate through aspartate-semialdehyde (ASA), so its synthesis adds to the amount of carbon withdrawn out of the tricarboxylic acid (TCA) cycle in the form of oxaloacetate (OAA). This carbon must then be replenished by anaplerotic reactions (Nelson et al., 2008), making the phosphoenolpyruvate-pyruvate-oxaloacetate (PEP-Pyr-OAA) node, already acknowledged as a major switching point for carbon metabolism (Sauer and Eikmanns, 2005), even more important for this organism.

**Figure 1:**
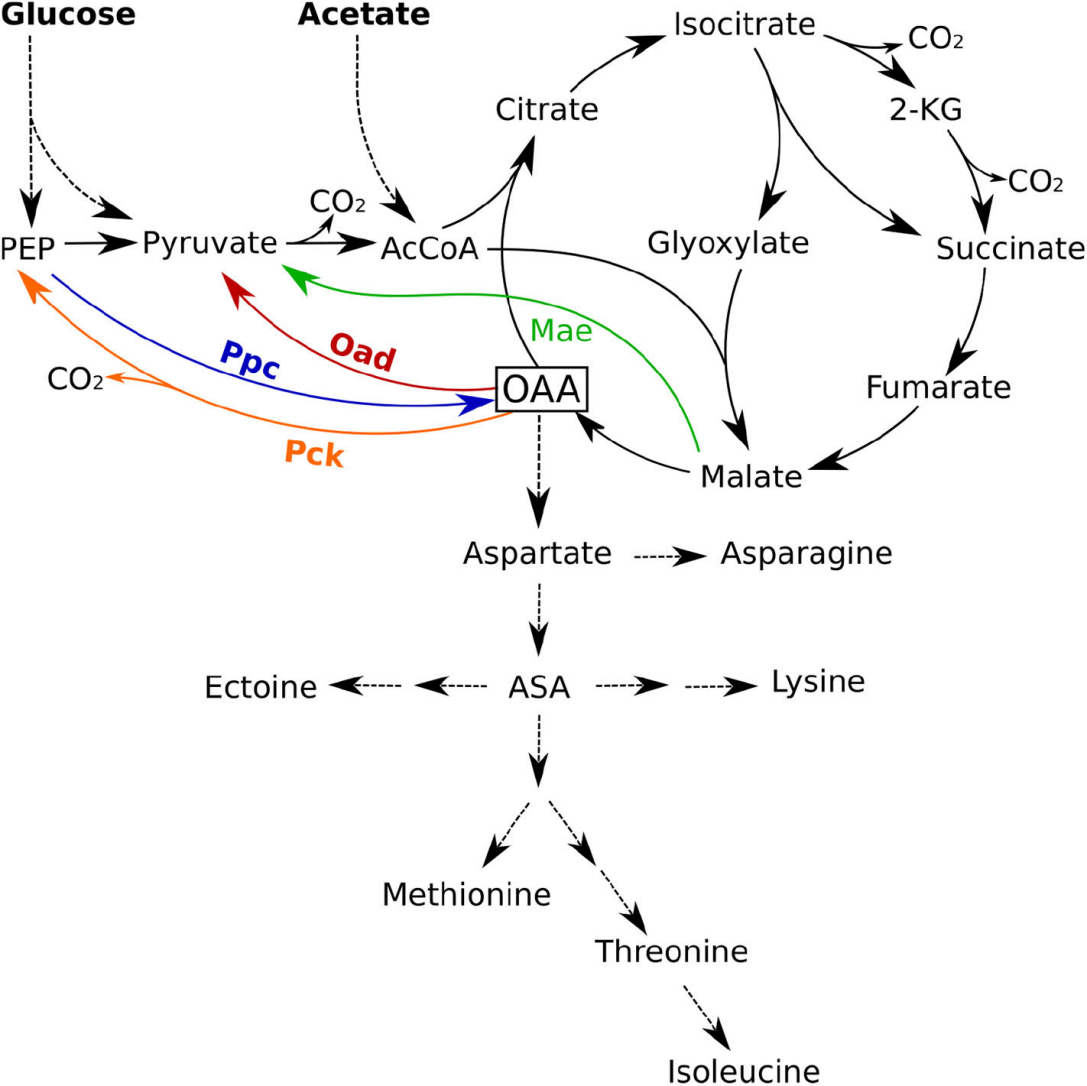
Anaplerotic reactions and TCA cycle in *Halomonas elongata*. Shown are the four enzymes phosphoenolpyruvate carboxylase (Ppc, blue), oxaloacetate decarboxylase (Oad, red), malic enzyme (Mae, green), and phosphoenolpyruvate carboxykinase (Pck, orange) that have the potential to catalyze anaplerotic reactions. ASA, Aspartate semialdehyde; 2-KG, 2-Keto-glutarate; AcCoa, Acetyl Coenzyme A.

Figure 1 shows the PEP-Pyr-OAA node and the glyoxylate shunt in *H. elongata* as it has been determined by genomic analysis (Schwibbert et al., 2011; Pfeiffer et al., 2017). The glyoxylate shunt bypasses the two decarboxylation steps of the TCA cycle and thus enables the replenishment of C4 carbon skeletons from acetyl coenzyme-A (acetyl-CoA). This pathway allows an efficient growth on acetate but its operation on glucose as a carbon source would result in a loss of one third of the total carbon in the decarboxylation of pyruvate to acetyl-CoA by pyruvate dehydrogenase. Anaplerotic reactions enable an efficient growth on hexoses by replenishing the carbon in the form of OAA produced directly from PEP or Pyruvate.

There are four enzymes that fulfill the stoichiometric requirements to catalyze anaplerotic reactions: phosphoenolpyruvate carboxylase (Ppc), malic enzyme (Mae), phosphoenolpyruvate carboxykinase (Pck), and the membrane bound oxaloacetate decarboxylase (Oad). The first of these enzymes, Ppc, has been known for a long time to carry most of the anaplerotic flux in *Escherichia coli* growing on glucose, glycerol or pyruvate (Ashworth and Kornberg, 1966; Peng et al., 2004) and its regulation has been shown to play a critical role in carbon catabolism (Xu et al., 2012). The second enzyme in the list, the malic enzyme (Mae), is present in two variants with different cofactor specificities (NADH and NADPH). The third candidate, phosphoenolpyruvate carboxykinase (Pck), is also an important enzyme in this node and catalyzes a key step for gluconeogenesis converting of OAA into phosphoenolpyruvate (PEP). Finally, Oad catalyzes the interconversion of OAA into pyruvate and deserves special attention in *H. elongata*. Cytoplasmic versions of the enzyme are considered to be irreversible, thermodynamically unable to function in the anaplerotic sense and usually involved in gluconeogenesis (Peters-Wendisch et al., 1997). In *H. elongata*, however, Oad is a membrane bound sodium pump (Dimroth et al., 2001) and it has been hypothesized (Kindzierski et al., 2017) that it can operate as an anaplerotic reaction, thus using the considerable sodium gradient available under high salt concentrations to drive the carboxylation of pyruvate in *H. elongata*. To the extent of our knowledge, such a function has never been observed *in vivo*. The different physiological roles described so far for Oad go in the opposite direction, for example, as a sodium-transport system energized by the decarboxylation of OAA into pyruvate (Dimroth, 1980, 1990, 1994; Dimroth and Schink, 1998) and as part of a catabolic pathway in a mutant strain of *Lactococcus lactis* (Pudlik and Lolkema, 2011).

The aim of this work is to shed light on how anaplerotic reactions are organized in *H. elongata* through a combination of theoretical and experimental methods. Due to their mechanisms, the action of two of these enzymes (Oad and Ppc) cannot be distinguished through labeling experiments (Pastor et al., 2013). Therefore, we constructed mutant strains lacking each of the enzymes (Ppc and Oad) and characterized their phenotypes in comparison to the wild type. The results of this experiments are examined under the light of thermodynamic analysis and simulations of constraint based models that are customized to deal with the challenge of estimating physico-chemical magnitudes at high salt concentrations.

## 2. Materials and Methods

### 2.1. Strains, Plasmids, and Growth Conditions

*H. elongata* DSM 2581^*T*^ and the mutant strains were routinely grown in minimal medium MM63 (Larsen et al., 1987) or LB medium (Miller et al., 1992) containing 1 M NaCl at 30°C under shaking at 220 rpm. All *Escherichia coli* strains were grown in LB medium at 37°C amended with the antibiotic necessary for maintenance of the plasmid. All plasmids and genetic manipulations were performed in *E. coli* DH5α or *E. coli* DH5α λ-pir.

### 2.2. Construction of Plasmids

Replacement of the *ppc* gene with *aadA*, encoding a streptomycin resistance cassette was performed by adapting the method previously reported for *Pseudomonas putida* (Mart́ınez-Garćıa and de Lorenzo, 2011). The plasmids were assembled by Gibson Assembly (New England Bioloabs, USA) according to the suppliers manual. Oligonucleotides used for the gene amplification are shown in Table 1. A DNA fragment containing about 500 bp of the genomic sequence upstream of the target gene (*ppc* or *oad*) followed by the sequence of the *aadA* gene, encoding the streptomycin resistance cassette, and about 500 bp of the chromosomal region downstream of the target gene, was inserted into pSEVA212S, carrying the R6K suicide origin of replication (Mart́ınez-Garćıa et al., 2015), which had been previously linearized with EcoRI (New England Bioloabs, USA). The plasmid was transformed (Chung et al., 1989) into *E. coli* DH5α λ-pir and clones carrying it were selected on LB agar plates supplemented with 200 μg/ml streptomycin (Sm 200). The correct assembly of pSEVA_Δ*ppc*::Sm and pSEVA_Δ*oad*::Sm was verified by isolation of the plasmids and sequencing (Eurofins Genomics, Germany).

**Table 1:** Oligonucleotides used in this work.

**Name**	**Sequence 5**′**–3**′****	**Function**
**Δ*****ppc*****::Sm**		
P_pSEVA212_F	atccccgggtaccgagctcgCACCCAACGAAAGCGAGGAGTCAC	^*^Construction of pSEVA_Δ*ppc*::Sm
P_up/aadA_R	gtcaaggttcTGCCCGAAGGGCCGGGCT	
P_up/aadA_F	ccttcgggcaGAACCTTGACCGAACGCAG	
P_down/aadA_R	agtctcgatcTTATTTGCCGACTACCTTGG	
P_down/aadA_F	cggcaaataaGATCGAGACTCTCCTTCTGAC	
P_pSEVA212_R	agggataacagggtaatctgAGACGCATCAGGTGCATTC	
gHe_P_F	GTGCTGTCGGTCAATACCCT	PCR and sequencing to verify mutant
gHe_P_R	CGCATTCCTCAGGACAACCT	
**Δ*****oad*****::Sm**		
O_pSEVA212_F	atccccgggtaccgagctcgGACCGAGCATGACATCGC	Construction of pSEVA_Δoad::Sm
O_up/aadA_R	tcggtcaaggttcGCGGTCTGCTCCTCGTTCG	
O_up/aadA_F	aggagcagaccgcGAACCTTGACCGAACGCA	
O_down/aadA_R	gccccatgcagggTTATTTGCCGACTACCTTGG	
O_down/aadA_F	agtcggcaaataaCCCTGCATGGGGCATCCTTG	
O_pSEVA212_R	agggataacagggtaatctgACATCTCGCTGGGCAACATC	
gHe_OAD_R	CCACGTCCCAGTCGATACAC	PCR and sequencing primer for
gHe_OAD_F	GTTCATCCACTGCTTTAGTC	verification of the mutant strain
gHe_OAD_F2	AATTGCAGGTGCACGGGA	sequencing primer
**General**		
pSW_F	GGACGCTTCGCTGAAAACTA	Verification of pSW-2 presence
pSW_R	AACGTCGTGACTGGGAAAAC	
ectC_F	ATCATCGAAACCAGCGGTCA	Verification of *H. elongata*
ectC_R	GCTGCGAACAACGAAAGAGC	
neo_F	CAGCGATCGTGTGTTTCGTC	Verification of pSEVA212S presence
neo_R	TTTGCAGGCTCGGGCTAAAT	

* *Sequences complementary to one fragment are shown in lower case and to the other in upper case. Supplier Eurofins Genomics, Germany*.

### 2.3. Conjugal Transfer of Plasmids Into *H. elongata* by Triparental Mating

Plasmids were transferred to *H. elongata* by a two step mating procedure. First, plasmid pSW-2 encoding the 1-SceI endonuclease (Mart́ınez-Garćıa and de Lorenzo, 2011) was transferred to *H. elongata*. Therefore, the donor strain *E. coli* DH5α λ-pir (pSW-2), the recipient strain *H. elongata*, and the helper strain *E. coli* HB101 (pRK600) were grown in LB medium, amended with the antibiotic necessary to assure plasmid maintenance (pSW-2: 10 μg/ml gentamycin and pRK600: 35 μg/ml chloramphenicol) until the stationary phase (over night). Optical densities at 600 nm (OD600) were measured and 1 ml of the donor culture was mixed with the amount of recipient and helper culture to obtain OD ratios of 1:1:1 and 1:2:2, respectively. These suspensions were centrifuged, the supernatant was discarded by decanting and the cell pellet was resuspended in the remaining medium rest. This condensed cell suspension was pipetted on one spot of a LB agar plate containing 0.5 M NaCl, and incubated for 5 h at 30°C. Afterwards, the cells were resuspended in 600 μl of a 1 M NaCl solution, an aliquot of 100 μl was plated on LB agar plates containing 0.5 M NaCl, 500 μg/ml ampicillin and 50 μg/ml gentamycin and the plates were incubated for 48 h at 30°C. Colonies were picked and the presence of pSW-2 was verified by PCR with pSW-F and pSW-R (Mart́ınez-Garćıa and de Lorenzo, 2011) (Table 1).

Next, pSEVA_Δ*ppc*::Sm and pSEVA_Δ*oad*::Sm were transferred and integrated in the genome of *H. elongata* (pSW-2) again by triparental mating. This was performed as described above but using *H. elongata* (pSW-2) as recipient, *E. coli* DH5α λ-pir (pSEVA_Δ*ppc*::Sm or pSEVA_Δ*oad*::Sm) as donor and *E. coli* HB101 (pRK600) as helper. The cointegrates were selected by plating different dilutions of the mating mixture on LB agar plates containing 0.5 M NaCl, 500 μg/ml ampicillin, 60 μg/ml kanamycin, and 50 μg/ml gentamycin to obtain single colonies.

### 2.4. Resolution of Cointegrates to Obtain Marker Substitution Mutants

To resolve the cointegrates a single colony was picked from the selective agar plate and grown in 3 ml LB medium with 1 M NaCl, 500 μg/ml ampicillin, 200 μg/ml streptomycin, and 50 μg/ml gentamycin over night at 30°C. The next day, cells were pelleted by centrifugation, washed once in LB medium containing 1 M NaCl, and subsequently resuspended to an OD600 of 1 with LB medium with 1 M NaCl, 500 μg/ml ampicillin, and 50 μg/ml gentamycin. To four ml of that culture, 80 μl of 3-methylbenzoate were added to a final concentration of 10 mM to induce the expression of 1-SceI and cells were incubated at 30°C under shaking.

After 3.5 h, an aliquot of cell suspension was plated on LB agar with 1 M NaCl, 500 μg/ml ampicillin, and 200 μg/ml streptomycin and incubated at 30°C for 48 h. Single colonies were picked and gridded on LB plates with 1 M NaCl, 500 μg/ml ampicillin, and either 60 μg/ml kanamycin or 200 μg/ml of streptomycin to identify those clones, that lost the kanamycin resistance cassette but maintained the streptomycin cassette. Clones showing the desired phenotype represent the desired mutants. This was verified by PCR using primers (gHe_P_F, gHe_P_R, gHe_OAD_F, gHe_OAD_R, gHe_OAD_F2; Table 1) that hybridized to sequences outside the 500 bp flanks used in pSEVA_Δ*ppc*::Sm or pSEVA_Δ*oad*::Sm and sequencing of the whole fragment.

The two mutant strains of *H. elongata* obtained by this procedure: Δ*ppc*::Sm and Δ*oad*::Sm will be named *H. elongata*-PPC and *H. elongata*-OAD, respectively.

### 2.5. Growth Experiments in Microplates

The growth experiments were performed in MM63 medium with 27.75 mM glucose or 27 mM acetate, respectively. Initially, a single colony of each strain was picked per replicate and grown in 3 ml LB medium with 1 M NaCl. The next day, an aliquot was used to inoculate 3 ml of MM63 medium with 1 M NaCl. From this culture, three aliquots were used to inoculate each of the pre-cultures for the growth experiment in MM63 medium with either 0.17, 0.5, 1, or 2 M NaCl. Inoculation was always done in a way to reach an initial OD600 of 0.01 and incubation was in all cases at 30°C in the shaker until the late exponential or early stationary phase. For the growth experiment, 200 μl of medium (MM63 with 0.17, 0.5, 1, or 2 M NaCl) were inoculated with 2 μl of the respective pre-culture adapted to the respective NaCl concentration in a sterile 96-well microtiter plate with optical bottom (Greiner, Germany) and incubated in an automated microplate reader (Tecan, Austria) at 30°C. Optical density was measured every 10 min after vigorous shaking for 3 s over a period of 16–22 h. In a first round of experiments, each condition was measured at least three times, i.e., starting with three different colonies per strain and per carbon source. The maximum growth rate of each well was defined as the maximum value of linear regression of the logarithm of the OD along a sliding window of at least 41 points. In order to ensure that the chosen values were part of a sustained exponential growth phase, only values that had a determination coefficient above 0.9 were considered eligible. Conditions that yielded significant differences in the first round of screening were analyzed in a second round using four biological replicates (from different colonies) each having two technical replicates (two wells inoculated from the same pre-culture). Differences reproduced in both experiments were tested in flasks.

### 2.6. Growth Experiments in Shake Flasks

Growth experiments in shake flasks were performed in MM63 medium with 27.75 mM glucose or 27 mM acetate, as carbon source in the presence of 0.17 M NaCl. Again, a single colony of the relevant strain was picked per replicate and grown in LB medium with 1 M NaCl. From these cultures aliquots were taken to inoculate the pre-cultures in MM63 medium with 0.17 M NaCl to an OD600 of 0.01. Pre-cultures of *H. elongata* wild type were incubated for about 12 h until they reached an OD600 of 1.5–2.6 on glucose and 0.5–0.8 on acetate, whereas pre-cultures of *H. elongata*-PPC were incubated for ~48 h to reach an OD between 4.9 and 6.0 in case of growth on glucose and between 1.0 and 1.2 in case of acetate grown cells. From theses pre-cultures pre-warmed 25 ml of MM63 medium with 0.17 M NaCl and either glucose or acetate as carbon source were inoculated to an OD600 of 0.05 and incubated under shaking for 11 h. The OD600 was measured every hour in a spectrophotometer (Eppendorf, Germany). The experiment was performed in triplicates, i.e., starting from three different colonies per strain.

### 2.7. Growth Experiments on Glucose or Acetate in Shake Flasks, Biomass, and Ectoine Quantification

The cultivations were performed in MM63 minimal medium with 27.75 mM glucose or acetate as carbon source in the presence of 2 M NaCl. For each strain (wild type, *H. elongata*-PPC and *H. elongata*-OAD) three single colonies were picked and grown in 1 M NaCl LB medium. From these pre-cultures aliquots were taken to inoculate cultures (Dötsch et al., 2008) to an OD600 of 0.01 in MM63 medium with 1 M NaCl and glucose (or acetate) as sole carbon source. This pre-culture step was then repeated once more with 2 M NaCl MM63 minimal medium. From these pre-cultures 50 mL of MM63 medium with 2 M NaCl and either glucose or acetate were inoculated to an OD600 of 0.01 and incubated under shaking 220 rpm for up to 70 h. The OD600 was measured regularly in a spectrophotometer (Eppendorf, Germany) until cells were harvested during the exponential phase. To release the intracellular ectoine a two-phase extraction protocol developed by Bligh and Dyer (1959) and further modified by Galinski and Herzog (1990) was applied. For this, the samples were centrifuged (15,000 g, 25°C) for 5 min and the supernatant was removed. The remaining cell pellet was resuspended in 250 μl extraction solution (methanol, chloroform, bidest. water in ratios 10:5:4). After an incubation period of 30 min under shaking (300 rpm, Thermomixer Comfort Typ 5, Eppendorf) at room temperature 65 μl chloroform and bidest. water were added and incubated a second time under shaking (300 rpm, Thermomixer Comfort Typ 5, Eppendorf, Germany) at room temperature for 10 min. To achieve the desired phase separation the samples were centrifuged once more at 8,000 × *g* for 5 min at room temperature. Then, 100 μl from the 250 μl aqueous upper phase were taken and diluted 1:10 in the mobile phase used for the subsequent high-performance liquid chromatography (HPLC) analysis. The ectoine content of the samples was measured by HPLC using a reverse-phase column (Nucleodur 100-5 NH2-RP CC 125/4, Macherey & Nagel, Germany) and applying an acetonitrile/phosphate buffer as mobile phase. The absorption of ectoine was recorded at 210 nm using an UV-detector (Kuhlmann and Bremer, 2002).

Biomass was quantified as ash-free dry weight (from now on DW) to prevent errors due to the variable salt content of the samples. Samples were taken at different values of OD600 harvested by centrifugation and washed using a 2 M NaCl solution. The pellets were freeze dried and analyzed gravimetrically with a simultaneous thermal analysis system (STA) (449C Jupiter, Netzsch Gerätebau GmbH, Germany) where the samples are heated on a precision balance. The weight loss and the heat transfer of the solid samples were recorded at a heating rate of 1°C min^−1^ (40–1,140°C) under a synthetic air (N_2_ and O_2_) atmosphere. The gas phase was analyzed by a mass spectrometry system (QMS 403 Aeolos, Netzsch Gerätebau GmbH, Germany). The following mass signals (amu) were recorded to identify decomposition fragments: 16 (O and NH_2_), 17 (OH and NH_3_), 18 (H_2_O), 30 (CH_2_-NH_2_), and 44 amu (CO_2_). The detection of CO_2_ by mass spectrometry was used to establish the temperature at which combustion started. Dry biomass samples always retain a certain amount of water and they tend to absorb more from the atmosphere in a time scale of minutes (Gurakan et al., 1990). This requires a desiccation process immediately before the measurements of biomass and ash. STA enables all these measurements to be part of a continuous process. Following heat flow and spectrometric data, it could be established that the mass loss up to 160°C is due to water. Since the DW correlates linearly with the OD600 of the culture, OD600 were measured and converted to DW using the calibration curve shown in the Supplementary Material.

### 2.8. Thermodynamics-Based Metabolic Flux Analysis (TMFA)

A simple stoichiometric model of the TCA cycle and the anaplerotic pathways in *H. elongata* was formulated based on previous stoichiometric models (Kindzierski et al., 2017). Stoichiometric information was complemented with additional thermodynamic information (Alberty, 2003; Flamholz et al., 2011). The stoichiometric and thermodynamic constraints were combined within the well establish framework of Thermodynamics-Based Metabolic Flux Analysis (TMFA) (Henry et al., 2007; Jol et al., 2010). This framework can be used to explore optimal modes of operation (flux distributions) of the metabolic network using Mixed Integer Linear Programming (MILP). Two modifications were introduced to the standard TMFA methodology. First, an additional constraint was added to ensure that the thermodynamic driving forces of all reactions were higher in magnitude than a pre-established limit. This becomes the Minimum Driving Force (MDF) as defined elsewhere (Noor et al., 2014). Second, a necessary step for thermodynamic calculations is the calculation of metabolite activities. The activity of a metabolite is a function of its concentration *a*_*i*_ = γ_*i*_*c*_*i*_ where γ_*i*_ is the activity coefficient. Activity coefficients in biochemistry are often calculated using the Debye-Hückel equation (Alberty, 2003), at *T* = 25°*C*.

(1) $$\begin{array}{l} {\text{log}}_{10}\gamma_{i}=-z_{i}^{2}\frac{0.509\sqrt{I}}{1+1.6\sqrt{I}} \end{array}$$

salt concentrations above 0.3 M, the D-H equations are no longer able to provide valid estimates for the activity coefficient because short range ion-specific interactions become significant, the effect of specific ion can be accounted for using methods, such as Specific ion Interaction Theory (SIT) (Guggenheim and Turgeon, 1955). This model requires the use of interaction coefficients for all the relevant ions *k* = 1…*N*_*k*_:

(2) $$\begin{array}{l} {\text{log}}_{10}\gamma_{i}=-z_{i}^{2}\frac{0.509\sqrt{I}}{1+1.5\sqrt{I}}+\underset{k}{\sum }\epsilon \left(j,k\right)x_{k} \end{array}$$

At *I* > 0.25*M*, we use equation (Guggenheim and Turgeon, 1955)

cations

(3) $$\begin{array}{l} \text{ln }\gamma_{i}=-\text{ln}\left(10\right)0.509z_{i}^{2}\frac{\sqrt{I}}{1+1.5\sqrt{I}}+\text{ln}\left(10\right)\epsilon \left(i,Cl^{-}\right)\;\left[NaCl\right] \end{array}$$

anions

(4) $$\begin{array}{l} \text{ln }\gamma_{i}=-\text{ln}\left(10\right)0.509z_{i}^{2}\frac{\sqrt{I}}{1+1.5\sqrt{I}}+\text{ln}\left(10\right)\epsilon \left(Na^{+},i\right)\;\left[NaCl\right] \end{array}$$

protons

(5) $$\begin{array}{l} \text{ln }\gamma_{H}=-\text{ln}\left(10\right)0.509\frac{\sqrt{I}}{1+1.5\sqrt{I}}+\text{ln}\left(10\right)\epsilon \left(H^{+},Cl^{-}\right)\;\left[NaCl\right] \end{array}$$

The most relevant coefficients in this case are ϵ(*Na*^+^, *Cl*^−^) = 0.03 and ϵ(*H*^+^, *Cl*^−^) = 0.12.

So every calculation involving extracellular species has to be performed according to SIT. The usual formula to transform energies of formation (Alberty, 2003), for instance, has to be corrected:

cations

(6) $$\begin{array}{l} \Delta_{f}G_{j}^{o^{\prime }}=\Delta_{f}G_{j}^{o^{\prime }}\left(I=0\right)+N_{H}\left(j\right)\;RT\text{ ln }\left(10\right)\;pH\\ \;-RT\;0.509\text{ ln }10\left(z_{j}^{2}-N_{H}\left(j\right)\right)\frac{\sqrt{I}}{1+1.5\sqrt{I}}\\ \;-RT\text{ ln }10\left[N_{H}\left(j\right)\epsilon \left(H^{+},Cl^{-}\right)-\epsilon \left(j,Cl^{-}\right)\right] \end{array}$$

anions

(7) $$\begin{array}{l} \Delta_{f}G_{j}^{o^{\prime }}=\Delta_{f}G_{j}^{o^{\prime }}\left(I=0\right)+N_{H}\left(j\right)\;RT\text{ ln }\left(10\right)\;pH\\ \;-RT\;0.509\text{ ln }10\left(z_{j}^{2}-N_{H}\left(j\right)\right)\frac{\sqrt{I}}{1+1.5\sqrt{I}}\\ \;-RT\text{ ln }10\left[N_{H}\left(j\right)\epsilon \left(H^{+},Cl^{-}\right)-\epsilon \left(Na^{+},j\right)\right] \end{array}$$

Once the model was formulated, TMFA simulations were performed at salt concentrations 0.17, 0.5, 1, and 2 M. For each case, ATP or OAA production were maximized in order to explore the potential theoretical yields. The simulations were repeated imposing the additional restriction that the minimal driving force (MDF) must be higher than different thresholds. We will designate the values of MDF using the notation Δ*G*_5_, Δ*G*_10_, and Δ*G*_95_ meaning the free energies associated to efficiencies of 5, 10, or 95%, respectively (Sehr et al., 2015). In other words, a flux distribution obtained for MDF = Δ*G*_99_ will have all its enzymes operating at a thermodynamic efficiency of at least 99% while in a flux distribution obtained for MDF = Δ*G*_01_, one or more enzymes can be operating only at 1% even at full saturation. The thresholds for MDF were chosen to ensure enzyme efficiencies ranging from 1 to 99% at the thermodynamic bottleneck. For simulations maximizing the yield of OAA, a minimum ATP production of 4.5 mmol/g DW was imposed to obtain a realistic split of the metabolic flux between OAA synthesis and catabolic TCA cycle of roughly 1:2 (Pramanik and Keasling, 1997). This choice does not affect the absolute differences in OAA yield between different solutions, but filters out wasteful solutions in terms of ATP. See Supplementary Material for more details.

## 3. Results

### 3.1. Ectoine Accumulation Dramatically Increases the Demand for OAA

The concentration of ectoine in the cytoplasm is known to be tightly regulated as a function of the salinity of the medium (Dötsch et al., 2008). Keeping the high concentration of ectoine that is necessary to balance the osmotic pressure of salt imposes the cost of maintaining a flux of ectoine synthesis equal to its dilution by growth. Thus, an exponentially growing culture must synthesize ectoine at a rate equal to its concentration times its growth rate. To evaluate the impact of ectoine synthesis on the central metabolic pathways, we have to compare the flux dedicated to ectoine production with other biosynthetic fluxes. As can be seen in Figure 1, ectoine is synthesized from aspartate; therefore its synthesis results in a demand for OAA in addition to that required for amino acid synthesis. The necessary data to estimate these fluxes is readily available in the literature. Growth rates and intracellular ectoine concentrations, have been consistently measured for the wild type (Dötsch et al., 2008), and it is widely assumed that the cell composition provided by Neidhardt and Umbarger (1996) is representative for gram-negative cells. Thus, the demand for OAA in *H. elongata* growing at different salinities can be estimated as explained in the Supplementary Material.

**Figure 2:**
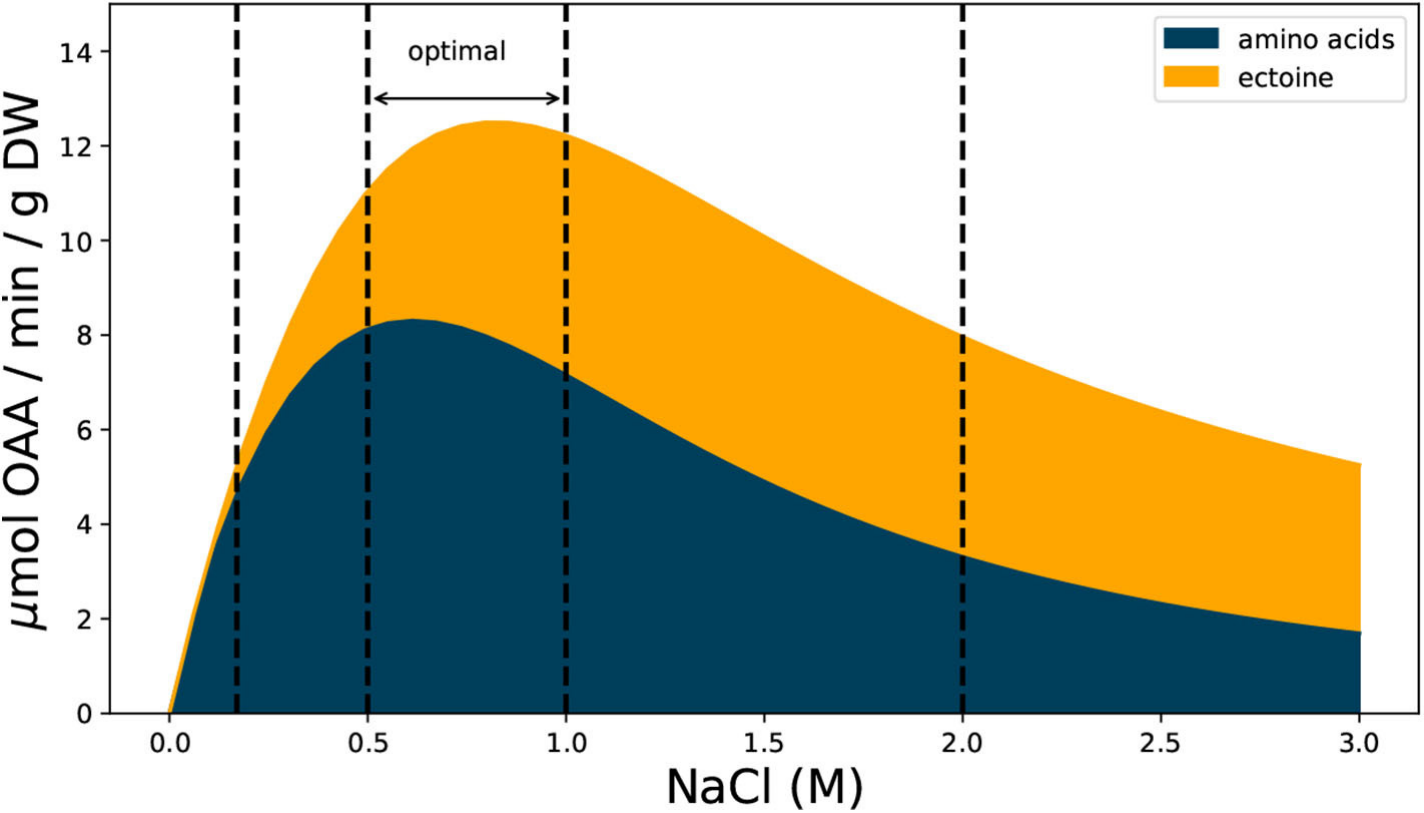
Estimated demand of OAA for ectoine and amino acid synthesis during exponential growth at different salt concentrations. The demand for amino acids is based on protein content and amino acid frequencies measured for *E. coli*. Ectoine demand based on reported values for *H. elongata* (see text and Supplementary Material for details). Vertical dashed lines mark salt concentrations of 0.17, 0.5, 1, and 2 M.

The estimate shown in Figure 2 reveals that the metabolic demand for OAA in *H. elongata* is similar to that of other bacteria at low salt concentrations (0.17 M NaCl) but the pattern quickly diverges as salt concentration increases. At the optimal interval for growth between 0.5 and 1 M NaCl, OAA demand reaches its peak and the cell dedicates up to 40% of the production to synthesize ectoine. As salt concentration increases even further and growth rates decline, the majority of OAA produced is destined to ectoine synthesis, thus keeping a high demand for OAA under growth rates at which other bacteria barely need any.

### 3.2. Only Ppc Is Thermodynamically Favorable, Oad Becomes Viable at High Salt

**Figure 3:**
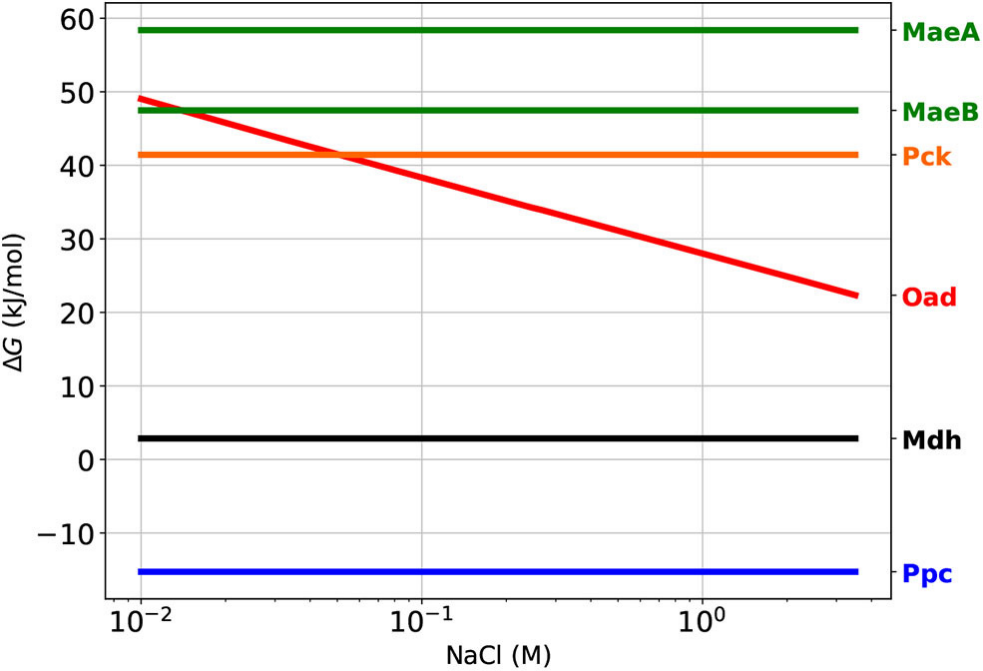
Thermodynamics of anaplerotic reactions. As a reference for *in vivo* conditions, cytoplasmic Na^+^ was fixed at 10 mM and external was varied from 0 to 3.5 M, CO_2_(total) = 10 μM P = 7.5 mM cofactor ratios NADH/NAD = 0.1; NADPH/NADP = 10; ATP/ADP = 10. All other metabolites were fixed at 1 mM. MaeA and MaeB are the NAP- and NADP-dependent malic enzymes, respectively. Ppc, PEP carboxylase; Oad, OAA decarboxylase; Pck, PEP carboxy-kinase. Malic dehydrogenase (Mdh) has been added for comparison since it is known to catalyze a thermodynamically challenging reaction (Noor et al., 2014).

In order to meet this higher than usual demand of OAA, *H. elongata* has four enzymes that can potentially catalyze anaplerotic reactions. Only one of these four enzymes, phosphoenolpyruvate carboxylase (Ppc), usually plays this role in other bacteria. The three remaining candidates, oxaloacetate decarboxylase (Oad), malic enzyme (Mae), and phosphoenolpyruvate carboxy-kinase (Pck) are normally considered to be thermodynamically unfavorable and thus not able to operate in the right direction within physiologically feasible concentrations of their reactants. In order to evaluate how the different environment and needs of *H. elongata* may lead to different roles for this enzymes, it is important to assess how these differences have an impact on the thermodynamics of the reactions involved. As discussed above, the high concentrations of electrolytes involved in the habitat of *H. elongata* result in solutions so far from ideality that the Debbye-Huckel corrections traditionally used in biochemical thermodynamics are insufficient. This obstacle was overcome by using Specific ion Interaction Theory (SIT) to account for the specific effects of Na^+^ and Cl^−^ ions on the activity coefficients, rather than using a generic correction based only on ionic strength. This is critical since for an enzyme to be able to operate *in vivo*, its free energy of reaction must be not only negative, but high enough in absolute value that it results in an efficient use of the enzyme (Noor et al., 2014). Standard free energies for the different reactions are often helpful to determine this, but can also be misleading in this case, since standard conditions do not account for gradients across the membrane. In Figure 3 we show orientative reference values for the free energies at different extracellular salt concentrations based on Equation (6) and the usual rules for thermodynamic calculations of reaction-transport processes (Jol et al., 2010). The salt dependence was calculated using physiologically realistic concentrations for intracellular sodium, phosphate and CO_2_, as well as realistic ratios for co-factors (Vojinović and von Stockar, 2009; Noor et al., 2014). All other substrates were assumed to be 1 mM. Of course, only the free energy of Oad changes with extracellular sodium concentration but the free energies of all potential anaplerotic enzymes are shown for comparison. Additionally a known thermodynamic bottleneck of the TCA cycle (Noor et al., 2014), malic dehydrogenase (Mdh), has been added as a reference. All free energies are calculated in the direction that leads to OAA production. The default anaplerotic reaction Ppc, shows an unsurprisingly negative free energy. Pck, which is known to be able to operate anaplerotically under extreme conditions (Zhang et al., 2009) albeit very inefficiently due to unfavorable thermodynamics, provides a counterexample with a very high, positive value. It is also noteworthy that both malic enzymes have even larger positive values, so they are very unlikely candidates for anaplerosis. Oad evolves from being very unfavorable in the absence of a sodium gradient to an enzyme able to work anaplerotically in saline environments. Interestingly, Oad is always less thermodynamically favorable than Ppc, regardless of the sodium gradient. Actually, even at high salt concentrations, Oad is still less favorable than Mdh.

It is noteworthy that all enzymes are reversible within physiological concentrations of their substrates, so there is no basis to discard any of them solely due to their thermodynamic properties.

### 3.3. Contributions by Pck and Mae to Anaplerotic Flux Are Severely Hindered by Distributed Thermodynamic Effects

**Figure 4:**
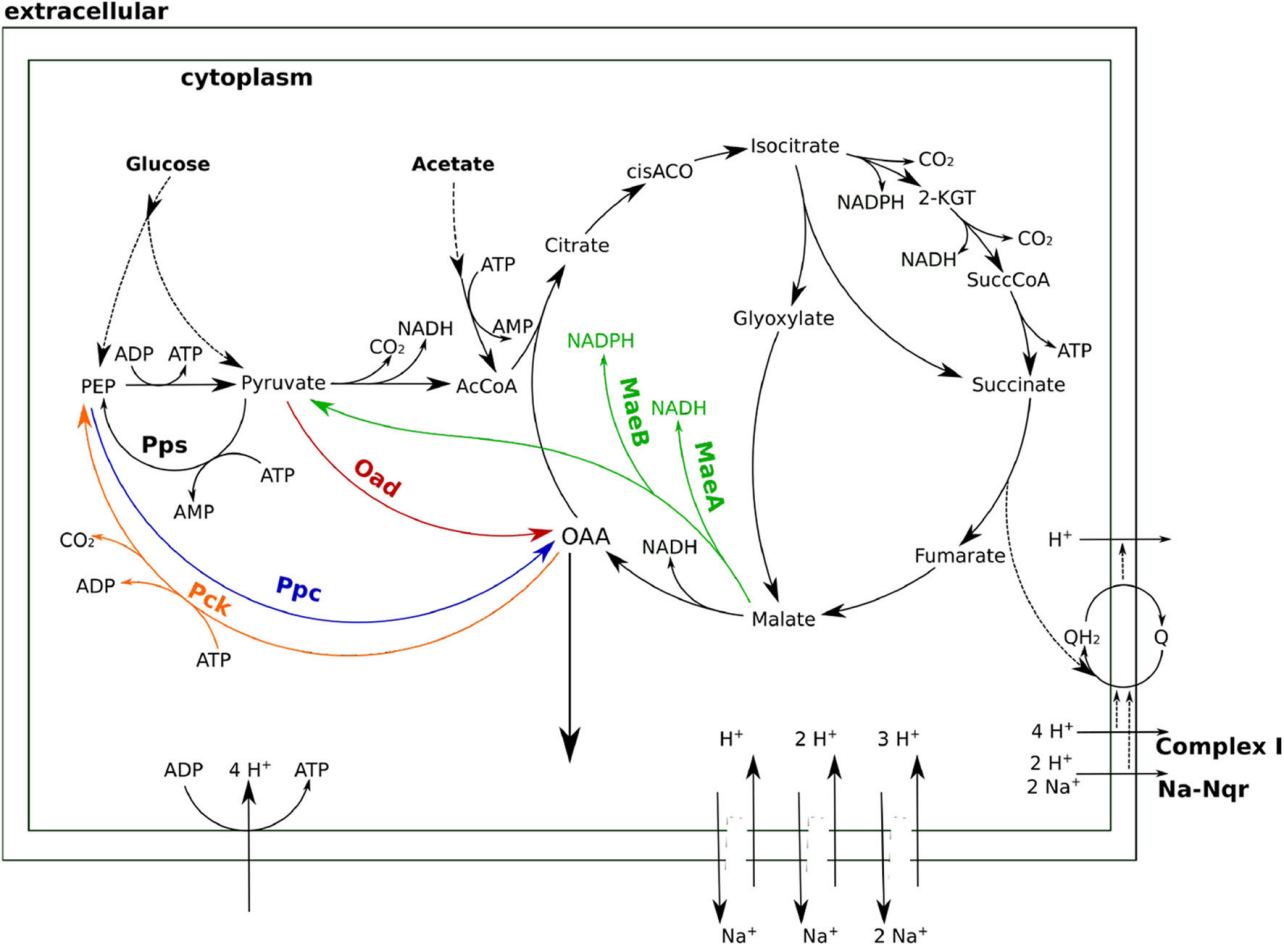
A stoichiometric model of TCA anaplerosis and related processes in *H. elongata*. Oad, OAA decarboxylase; Ppc, PEP carboxylase; Pck, PEP carboxy-kinase; MaeAB, malic enzyme A and B; Pps, PEP synthase; Na-Nqr, ubiquinone oxidoreductase.

Although all four enzymes can operate in the right direction when working in isolation, it remains to be seen whether they can carry the flux *in vivo*. In order to combine the stoichiometric and thermodynamic aspects in an integrated analysis of the relevant pathways, a model based on stoichiometric and thermodynamic constraints was formulated (see Supplementary Material). Although focused on the TCA cycle and the anaplerotic reactions, this model includes other relevant processes, such as the respiratory chain, ATPase and other processes related to sodium transport. As can be seen in Figure 4, sodium plays multiple roles in this metabolic network beyond driving Oad. The electron transport chain of *H. elongata* has a sodium translocating NADH ubiquinone oxidoreductase (Na-Nqr) (Kindzierski et al., 2017) which couples respiration to the sodium export. Moreover, as many other halophilic and halotolerant bacteria, *H. elongata* has a high number of sodium-proton antiporters that can be used to built a proton gradient or to use one to pump sodium out of the cell. The different stoichiometries of the antiporters provide different degrees of coupling between proton and sodium transport just like the gears in a gearbox.

Calculating the optimal flux distributions to maximize the production of OAA points at Pck and Mae as the most advantageous anaplerotic reactions from a stoichiometric point of view, as has already been pointed out (Schwibbert et al., 2011; Kindzierski et al., 2017). In spite of their reversibility within the established *in vivo* ranges for metabolite concentrations no thermodynamically viable flux distributions could be obtained where Pck or the malic enzymes played an anaplerotic role. In order to operate in the carboxylating direction, these enzymes require concentration ratios between their substrates and products that are incompatible with the requirements of other reactions in the network. This can be clearly seen for the malic enzyme since the low malate concentration needed to enable anaplerotic flux through Mae is incompatible with the high concentration required to drive the next enzyme in the sequence, Mdh, toward OAA production.

It is worth mentioning that computer simulations of this system often exhibit the simultaneous action of both malic enzymes in opposite directions, which results in a net transfer of electrons from NADPH to NADH. These chemically feasible solutions point to the risk faced by the cell if these two enzymes were simultaneously active. Only a strict control of the expression and/or activity of this enzymes can prevent them from destroying the carefully balanced redox state of the two electron currencies in *H. elongata*. Enzyme assays with cell free extract have shown very low activities for Mae using NADP as cofactor (Kindzierski et al., 2017). No activity could be detected using NAD (personal communication, Irina Bagyan).

### 3.4. Ppc Is Stoichiometrically as Inefficient as the Glyoxylate Shunt

**Figure 5:**
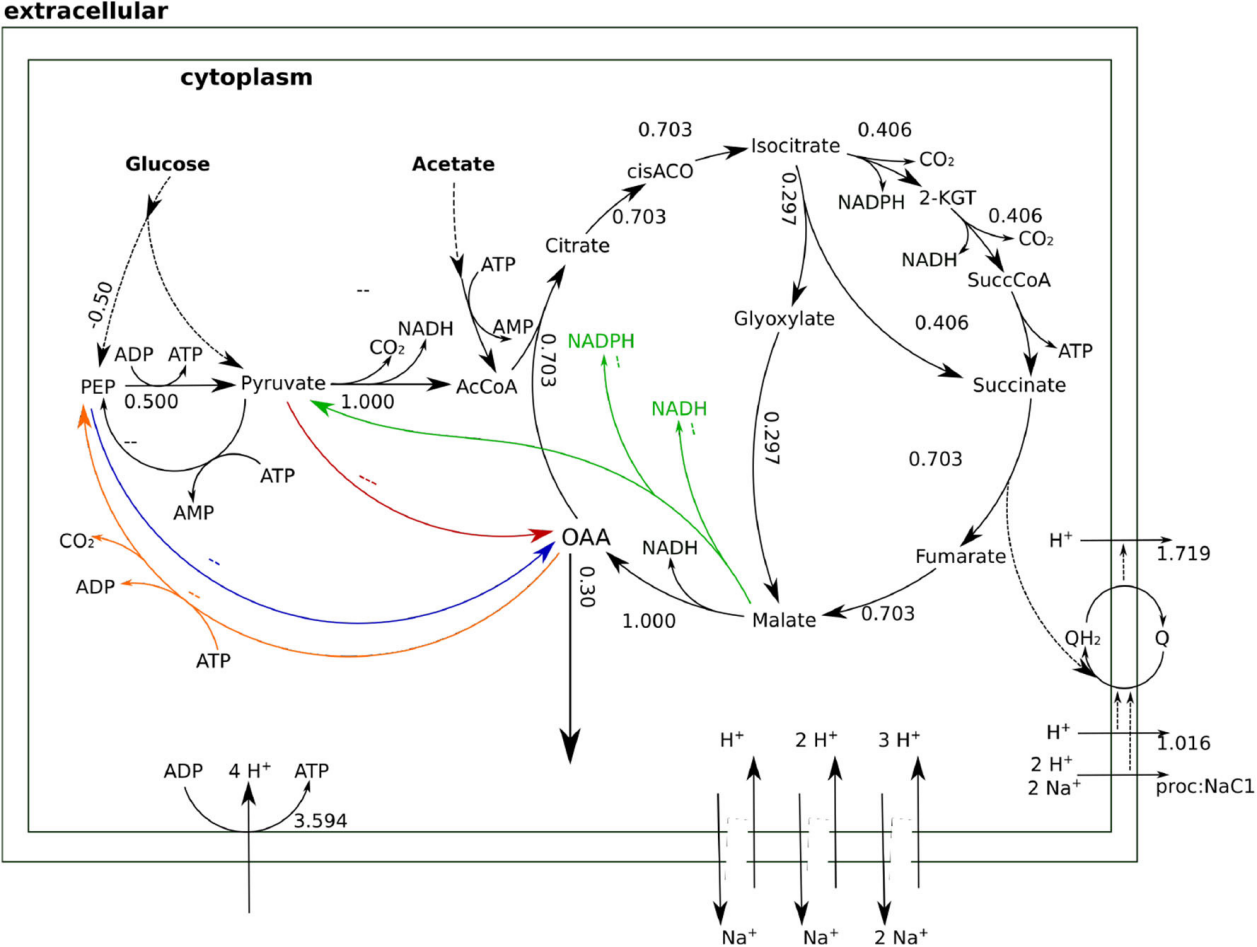
Flux distributions for optimal OAA yield with Oad inactive indicated on the layout of Figure 4. All fluxes are normalized per unit of glucose input. In this flux distribution, the glyoxylate shunt prevents a fraction of the carbon flowing into the TCA cycle from being lost as CO_2_, thus enabling a net production of OAA in the cycle.

**Figure 6:**
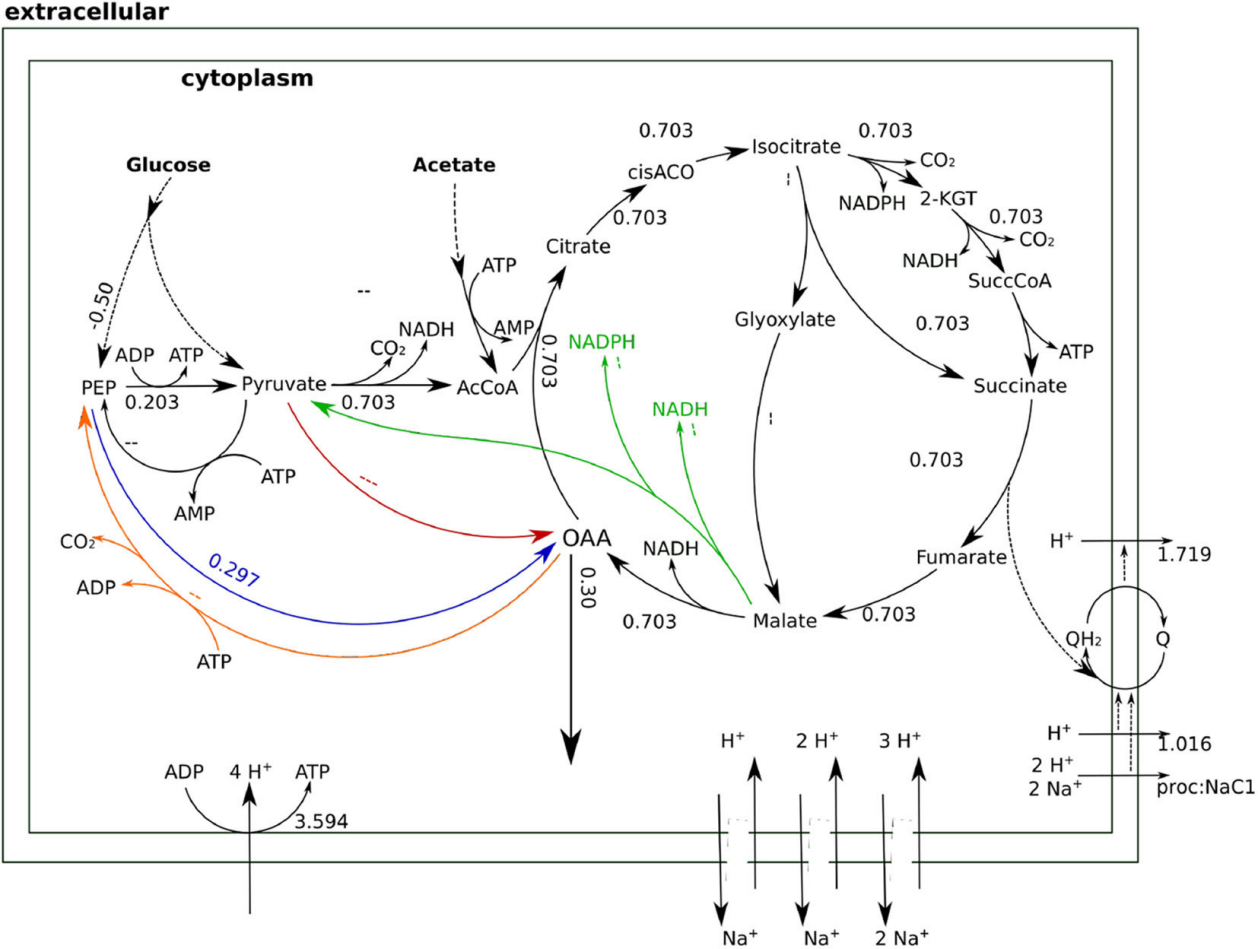
Flux distributions for optimal OAA yield with Oad inactive indicated on the layout of Figure 4. All fluxes are normalized per unit of glucose input. This flux distribution channels anaplerotic flux through Ppc. The need to produce ATP to convert PEP into pyruvate leads to a higher catabolic flux in the TCA cycle that results in some carbon being channeled to the catabolic part of the TCA cycle.

The type of glycolysis presumed to be active in *H. elongata* is the Entner-Doudoroff (ED) pathway (Klingner et al., 2015; Kindzierski et al., 2017). This pathway splits glucose into pyruvate and PEP in a 1:1 proportion which poses a difficulty to channel flux through Ppc. Recovering PEP for anaplerosis would require phosphorylating pyruvate back to PEP. This is a difficult reaction that consumes two ATP equivalents and reduces the OAA yield due to the need to increase metabolic flux to catabolism for ATP production. This can be seen quantitatively by blocking all sodium related processes in the model, and therefore Oad, and then calculating the optimal flux distribution for OAA production. This simulation results in two equivalent solutions. On the one hand, the flux can proceed through the glyoxylate shunt as shown in Figure 5. The loss of one carbon in three in the decarboxylation of pyruvate to Acetyl-CoA, results in a low yield of 0.3. On the other hand, the flux distribution shown in Figure 6 avoids the decarboxylation step but requires a higher ATP production due to the need to produce PEP from pyruvate, which results in exactly the same low yield. Thus, having Ppc as the only anaplerotic enzyme is stoichiometrically equivalent to having no anaplerotic enzymes at all.

**Figure 7:**
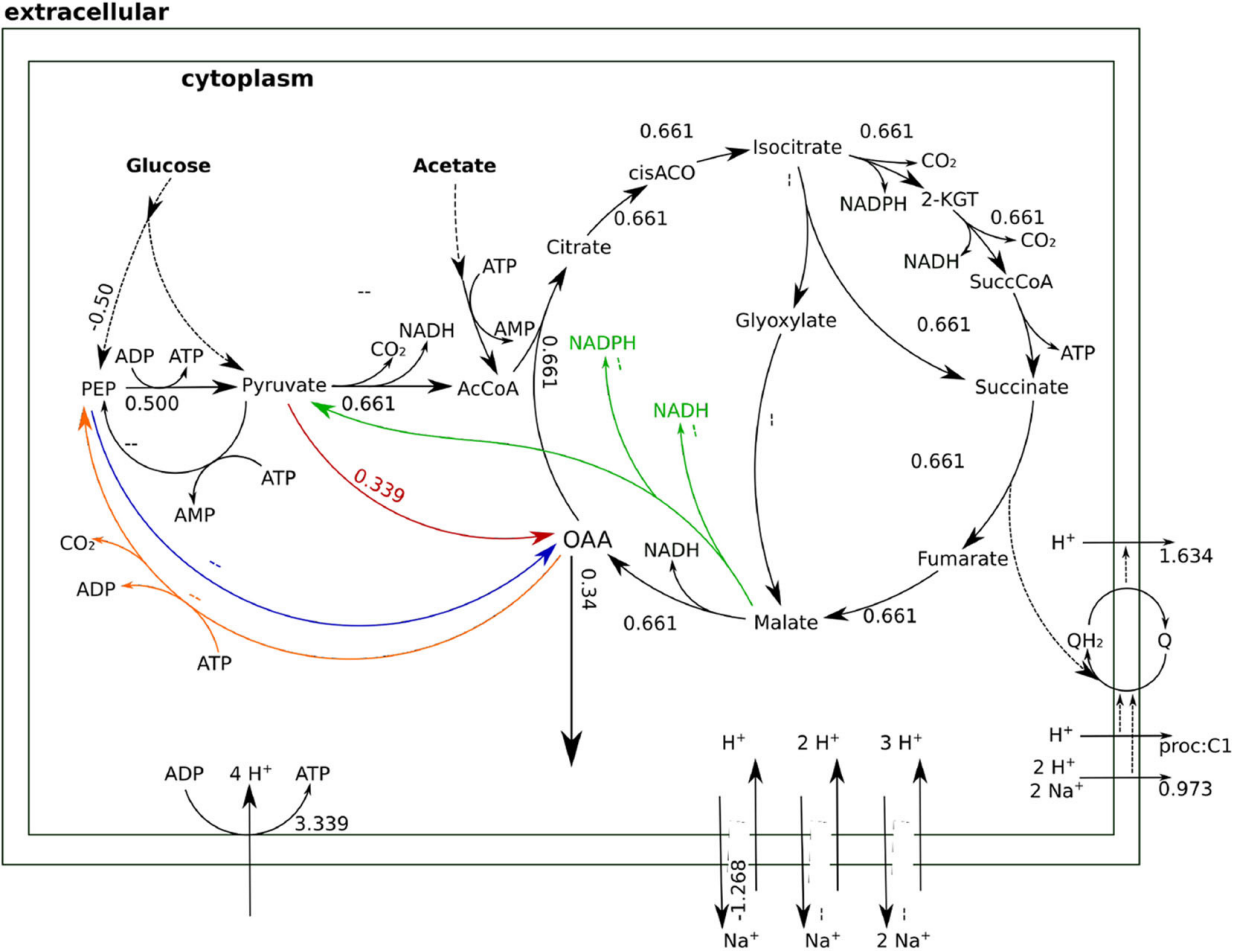
Flux distributions for optimal OAA yield at 1 M NaCl indicated on the layout of Figure 4. All fluxes are normalized per unit of glucose input. In this flux distribution, Oad carries the anaplerotic flux achieving a higher OAA yield.

Whenever the sodium gradient allows it, Oad can carboxylate pyruvate directly to obtain higher yields of OAA as shown in Figure 7. Beyond providing a stoichiometrically efficient solution, Oad ties central metabolism together with the respiratory chain and membrane transport systems into the sodium economy in the cell. The sodium influx through Oad can be a counterbalance to the sodium export by the ubiquinone oxidoreductase (Na-Nqr) or, when there is a net influx of sodium, be compensated with the Na^+^/H^+^ antiporters or ATP dependent Na^+^ exporters.

### 3.5. Anaplerotic Flux Is Distributed Between Ppc and Oad With a Sodium Dependent Ratio

In order to establish the roles of Ppc and Oad, growth experiments were done with the two mutant strains described above: *H. elongata*-PPC and *H. elongata*-OAD. The phenotype associated to the deletions was characterized by cultivating the wild type *H. elongata* and each of the mutants at different salt concentrations on both glucose and acetate. Growth on acetate here serves as a control in which anaplerotic flux becomes irrelevant because the incorporation of carbon into the TCA cycle occurs through the glyoxylate shunt. Therefore, any phenotype caused by an insufficiency of the anaplerotic reactions, should disappear when cells grow on acetate.

**Figure 8:**
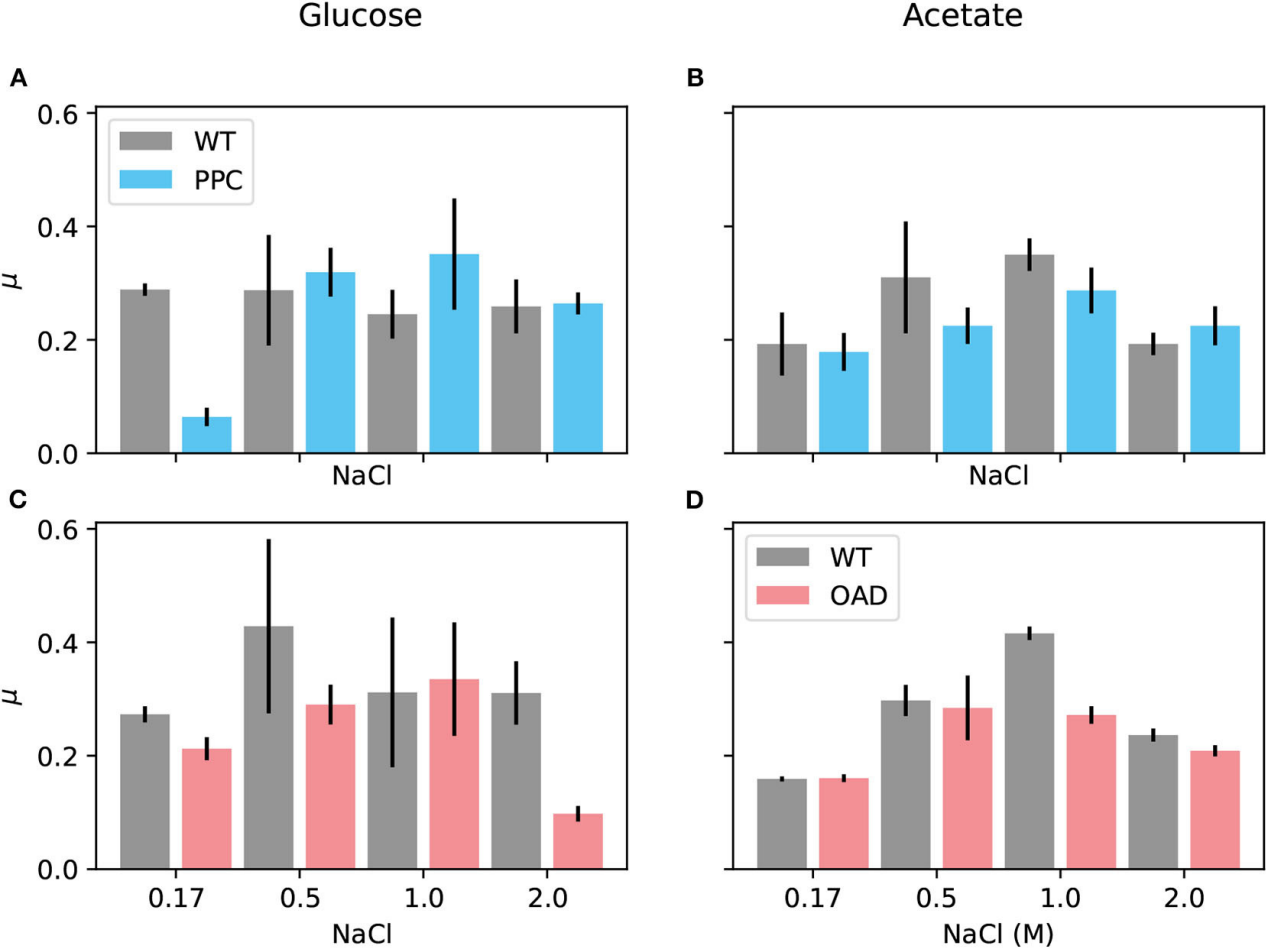
Growth of *H. elongata* on acetate. Cells of each of the mutant *H. elongata* strains were grown at 30°C in mineral salt medium (MM63) with NaCl (salt) at a concentration of 0.17, 0.5, 1, and 2 M, respectively, and with glucose (**A,C** on the left) or acetate (**B,D** on the right) as sole carbon source. Each of the experiments compared maximum growth rates of a mutant strain (*H. elongata*-PPC in blue and *H. elongata*-OAD in red) in comparison to the wild type (WT black).

A first screening using a microtiter plate reader enabled to test a wide variety of media spanning salt concentrations from 0.17 to 2 M for each strain growing on glucose and acetate. Figure 8 summarizes the maximal growth rates observed in one such experiments. Inspection of the figure and the growth curves for this and similar experiments (all collected in the Supplementary Material) draws attention to two conditions that lead to very clear phenotypes: First, *H. elongata*-PPC at low salt (0.17 M NaCl) grows as well as the wild type on acetate (Figure 8B) but it grows much slower on glucose (Figure 8B). Second, *H. elongata*-PPC at high salt (2 M NaCl) also shows normal growth on acetate (Figure 8D) while having difficulties on glucose (Figure 8C).

A selection of the experimental setups surveyed in the microtiter plate experiments, including the two cases already mentioned, was analyzed in shake flask experiments that allow a more accurate and reproducible quantitative treatment (see Supplementary Material). In most cases, the flask experiments yielded a more accurate picture of what was already indicated by the previous screening. *H. elongata*-PPC growing in a medium with 0.17 M salt exhibited similar growth rates as the wild type when growing on acetate (0.16 ± 0.02 *h*^−1^ vs. 0.19 ± 0.004 *h*^−1^) but experienced difficulties when growing on glucose (0.06 ± 0.04 *h*^−1^ vs. 0.28 ± 0.007 *h*^−1^). Comparison of growth rates on glucose at high salt, however, provide a finer comparison between the three strains. Besides the dramatic drop from a growth rate of 0.25 ± 0.01 *h*^−1^ in the wild type to 0.09 ± 0.004 *h*^−1^ in *H. elongata*-OAD, a smaller decrease to a growth rate of 0.13 ± 0.01 *h*^−1^ is shown in the Ppc lacking strain. Increased lag phases for both mutant strains on glucose were often observed in the pre-cultures, especially during growth in their respective inhibitory salt condition. Furthermore, the deletion of *ppc* seems to have a higher impact on metabolism characterized by higher fluctuations in growth rate. This can be seen in the growth data of the flask experiments shown in the Supplementary Material where exponential phases for the wild type and *H. elongata*-OAD could be fitted with determination coefficients of 0.99 while those for the *H. elongata*-PPC strain could be as low as 0.8.

### 3.6. Robustness of Ectoine Levels at High Salt

**Figure 9:**
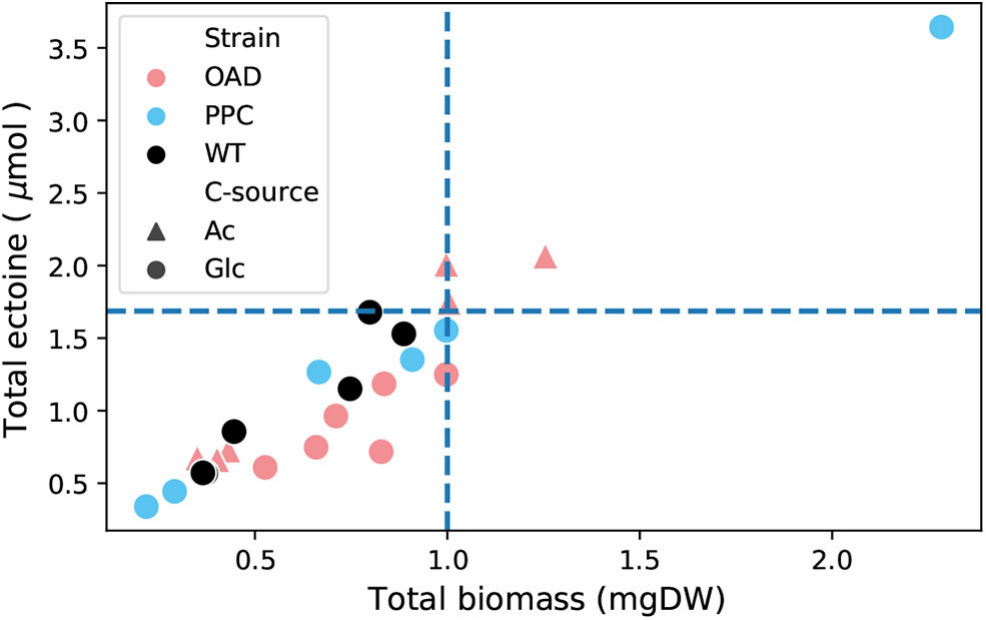
Biomass vs. ectoine for different strains of *H. elongata*: WT, OAD, and PPC growing on glucose (Glc) and acetate (Ac). The data includes samples taken at two different time points from three cultures for each combination of strain and carbon source. Dashed lines show the predicted expected ectoine content calculated from data available in the literature (Dötsch et al., 2008).

The ectoine content was determined in cells of *H. elongata* wild type and the two mutant strains *H. elongata*-PPC and *H. elongata*-OAD growing at high salt (2 M NaCl). Cells growing at low salt (0.17 M NaCl) were not included in this analysis because under such conditions the ectoine concentration is too low to be measured reliably. Figure 9 shows the ectoine content of the samples against their biomass. The linear trend is quite consistent regardless of strain or carbon source and also agrees well with values reported in the literature. Only *H. elongata*-OAD growing on glucose consistently show lower ectoine contents. This strain grew roughly 30% slower (μ = 0.09 *h*^−1^) in such conditions compared to *H. elongata* wild type (μ = 0.25 *h*^−1^).

**Figure 10:**
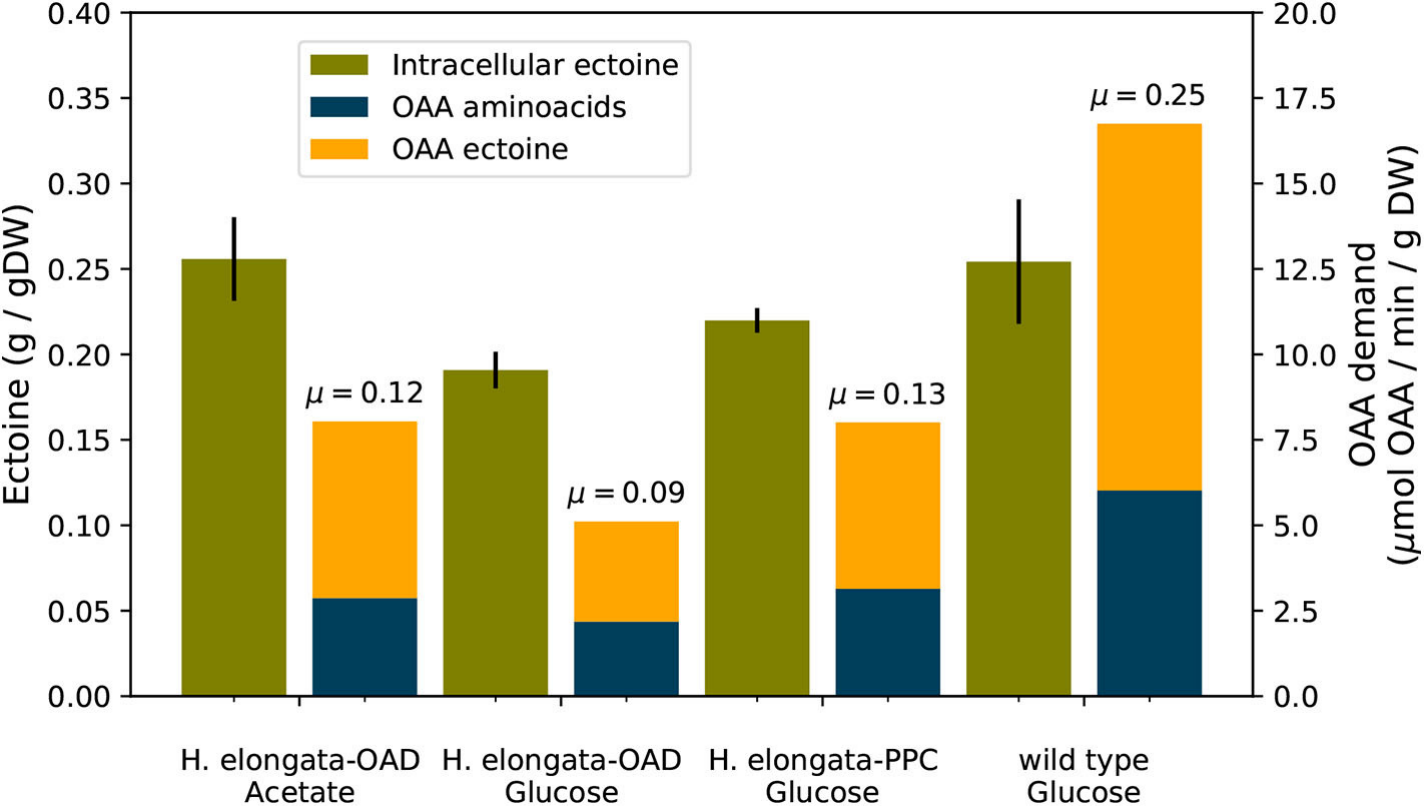
Ectoine content (•) and OAA demand for amino acid (•) and ectoine synthesis (•) in different strains at 2 M salt. Growth rates indicated on top of OAA demand bars. The growth rates were calculated from the shake flask experiments described above. A table summarizing these results including standard deviations can be found in the Supplementary Material.

The experimental data shown in Figure 10 can be used to estimate the demand of OAA for these strains as was done in the previous section. Without Oad the anaplerotic flux reaches only 30% and without Ppc only 50% of the flux calculated for the wild type employing both, Oad and Ppc. These results point to a shared anaplerotic role between Oad and Ppc even at fairly high salt concentrations.

**Figure 11:**
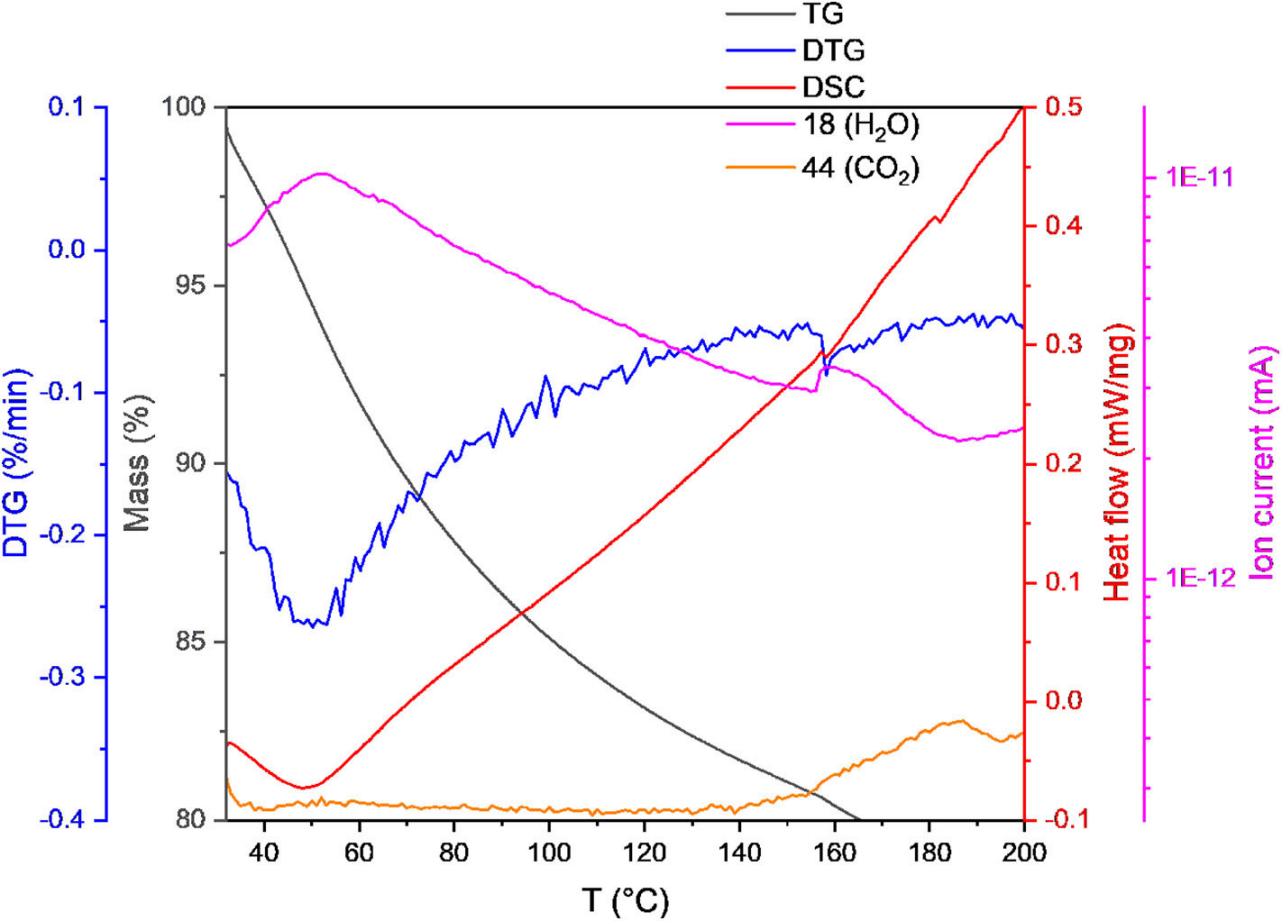
Simultaneous thermal analysis of dry weight from *H. elongata* (OD 1.2). The mass is detected with a precision balance over a temperature range from 32 to 200°C with a heating rate of 1 K/min under air atmosphere. The heat uptake is compared to an empty crucible and the heat flow is plotted over the whole temperature range. Furthermore, different mass traces corresponding to water (18) and CO_2_ (44) are recorded for the decomposition process with a quadrupole mass spectrometer which is coupled to the STA (see Supplementary Material for graph over the full temperature range).

Accurate determination of the biomass in terms of ash-free dry weight was made possible by using simultaneous thermal analysis (STA) up to a temperature of 1,140°C. Figure 11 shows an extract of the data for a sample up to 200°C, the full curve can be seen in the Supplementary Material. At lower temperatures, the heat flow is negative due to the predominance of endothermic processes. For all temperatures below 160°C the mass spectrometry detects the presence of water (mass 18 Da) and the absence of CO_2_ (44 Da). This temperature is taken as the initiation point for decomposition due to the appearance of CO_2_ in the gas phase. Thus, the loss of mass before reaching 160°C is taken to be the evaporation of the moisture in the sample (Gurakan et al., 1990) and the mass remaining at the end of the process is the ash content, with any loss in-between being the proper biomass. The water content of the samples was between 6 and 10%.

## 4. Discussion

The need to synthesize ectoine alters the metabolic demand for OAA in *H. elongata*. This demand is not only higher but also tuned to different stimuli. While the demand for OAA in similar bacteria is directly proportional to the growth rate, that of *H. elongata* also depends on the salinity of the medium. Growth rates at 0.17 M NaCl and 2 M NaCl, for instance, are very similar but as can be seen in Figure 2, the demand for ectoine is noticeably higher in the second case. Different needs lead to different selection pressures that condition how this organism produces OAA. The results of this study clearly indicate that Oad can operate as an anaplerotic enzyme as was previously hypothesized by Kindzierski et al. (2017). Moreover, the anaplerotic flux is shared between this enzyme and Ppc under a wide range of salt concentrations. Ppc is thermodynamically more favorable than Oad but this comes at the cost of lower stoichiometric yields. When the salt concentrations is high enough, Oad can carry most of the necessary flux for normal growth and ectoine production, even in the absence of Ppc. The difficulties of the strain *H. elongata*-OAD to grow at a salt concentrations of 0.17 M, show that Oad can no longer operate efficiently at this concentration. For the model simulations to reflect this phenotype, it is necessary that the minimum intracellular sodium concentration be fixed at 10 mM. Allowing lower values results in simulations where Oad can operate efficiently under such conditions.

The intracellular sodium concentration is the variable through which the cell can modulate the sodium gradient to a certain extent. The architecture of the metabolic network implies that the sodium concentration gradient across the membrane must respect a balance between two opposing forces. It must be steep enough to let Oad operate anaplerotically but not so steep that it prevents the correct operation of the electron transport chain. But cytoplasmic sodium is difficult to measure, due to the impossibility to distinguish between its free and bound forms. Total sodium in *E. coli* has been reported to be 5 mM (Shabala et al., 2009; Milo et al., 2010) while for *H. elongata* a wide range of values has been reported between 40 and 630 mM for extracellular salt concentrations between 0.05 and 3.4 M (Vreeland et al., 1983). Free sodium at high concentration exerts a cytotoxic effect so it is to be expected that a large fraction of this sodium is bound to cell structures. This is supported by the fact that some of this structures become more acidic at higher salt concentrations to increase its binding capacity for cations (Vreeland et al., 1984). This is indeed an issue of importance for the survival of the cell, which has developed methods to protect itself against excessive sodium concentrations. *H. elongata* and other halophilic bacteria are equipped with a number of different sodium efflux pumps. In *H. elongata* at least 10 different genes and gene clusters, respectively, can be found that code for putative sodium exporters belonging to seven different transporter families (Schwibbert et al., 2011; Pfeiffer et al., 2017). The sodium export transporters comprise various Na^+^/H^+^ antiporters (NahA, NahC, NahP), a respiratory-dependent sodium translocating NADH ubiquinone oxidoreductase (Na-Nqr) and a H^+^ transporting two sector ATPase. In comparison, *E. coli* has only four systems to extrude sodium, namely NahA, NahB, ChaA, and a respiratory-dependent exporter (Shijuku et al., 2002).

But extrusion is not the only mechanism available to *H. elongata* to protect its cytoplasmic interior against the adverse effects of sodium. Compatible solutes like ectoine are considered water-structure makers or kosmotropes, while ions like Na^+^ are water-structure breakers or chaotropes. Kosmotropes are often seen as stabilizers of biological molecules (for instance proteins) and chaotropes as their denaturants (Baldwin, 1996; Galinski et al., 1997; Zhang and Cremer, 2010). Employing Raman spectroscopy, (Hahn et al., 2016) could show that ectoine enhances water populations with stronger hydrogen bonding, while sodium is increasing water populations with weaker hydrogen bonding. The formation of strongly bound water around ectoine totally compensate the effect of sodium up to a salt concentration that was 40% of the ectoine concentration. Because of this behavior it may be justify calling ectoine a “NaCl compensatory solute.” All in all, the combination of simulations and indirect experimental evidence seems to back up the idea that intracellular free sodium concentration in the cytoplasm of *H. elongata* may be higher than that of other gram-negative bacteria. For now, no exact value can be given for these concentrations but the range shown by the model provides a ballpark estimate that is coherent with the information we have.

A coexistence of parallel pathways as happens with the anaplerotic reactions described in this study, is frequent among bacteria (Sauer and Eikmanns, 2005). This can be understood in evolutionary terms as a source of robustness through redundancy, a way to ensure flexibility by providing alternatives that work well under different conditions. An example of this are the high and low affinity pathways for ammonia assimilation, which guarantee an efficient incorporation of nitrogen into amino acids regardless of the concentration of ammonia (van Heeswijk et al., 2013; Sehr et al., 2015). But metabolic alternatives can also be a regulatory necessity as is the case with some isoenzymes. The parallel operation of several enzymes at the beginning of a branched pathway, e.g., amino acid synthesis—enables the independent inhibition of each by a different end-product. The unusual architecture of the anaplerotic reactions in *H. elongata* seems to obey to a combination of all of the above. Ppc and Oad offer alternative pathways to guarantee an anaplerotic flux under different conditions. That these conditions overlap is shown by the fact that a mutant lacking either enzyme can achieve the same growth rate as the wild type at some salt concentrations. Moreover, as this work shows, low salt concentrations render Oad as an inviable or very inefficient option, so the presence of Ppc widens the range of salinities in which *H. elongata* can thrive. Finally, the anaplerotic reactions are a critical part of two different metabolic functions providing the basis for both amino acid synthesis and salt tolerance. The environmental factors that condition these two functions can be independent from one another, e.g., a change in carbon source would result in a large change in amino acid demand for growth but would not alter the set concentration for ectoine. The coexistence of different enzymes enables a more flexible regulatory scheme where different enzymes can be modulated by different factors. The sodium dependence of Oad guarantees an increase in anaplerotic flux to support increased ectoine production at higher salt while Ppc can respond to an increased demand of OAA for amino acid biosynthesis. Further investigations on this topic will surely provide valuable insights and improve our understanding of the design principles that shape this part of bacterial metabolism.

## Data Availability Statement

The raw data supporting the conclusions of this article will be made available by the authors, without undue reservation.

## Author Contributions

AM-S, HK, SB, and AK obtained the funding. HK and AM-S conceptualized the research. AM-S and KP-G supervised the work. KH, MG, and SS carried out the experiments. CS and AM-S wrote the software and conducted the computer simulations. AM-S wrote the first draft of the manuscript. KH, KP-G, and HK participated in the elaboration of subsequent versions. All authors read and approved the final version of the manuscript. All authors contributed to the article and approved the submitted version.

## Conflict of Interest

The authors declare that the research was conducted in the absence of any commercial or financial relationships that could be construed as a potential conflict of interest.
