# Supplementary material for: Anaplerotic Pathways in Halomonas elongata: The Role of the Sodium Gradient
Source: Front Microbiol. 2020 Sep 25;11:561800. doi: 10.3389/fmicb.2020.561800 (PMC7545133; doi:10.3389/fmicb.2020.561800)
Supplement: Supplementary file 1 [file Data_Sheet_1.PDF]

# Supplementary Material

## 1 CALCULATING THE METABOLIC DEMAND FOR OXALOACETATE

Gram-negative cells are commonly assumed to have a very similar composition to that of *E. coli* as published by Neidhardt (Neidhardt and Umbarger, 1996). This reference estimates the protein content of the cell as 55% of the total dry weight with the following distribution of frequencies among the different amino acids.

This enables to estimate the content of each of the amino acids synthesized from oxaloacetate in millimoles per g of dry weight. Assuming an average amino acid molecular weight of 110 g/mol:

| | acronym | name | molar fraction |
|---|---|---|---|
| A | ala | L-alanine | 0.096 |
| R | arg | L-arginine | 0.055 |
| D | asp | L-aspartate | 0.045 |
| N | asn | L-asparagine | 0.045 |
| C | cys | L-cysteine | 0.017 |
| E | glu | L-glutamate | 0.049 |
| Q | gln | L-glutamine | 0.049 |
| G | gly | glycine | 0.115 |
| H | his | L-histidine | 0.018 |
| I | ile | L-isoleucine | 0.054 |
| L | leu | L-leucine | 0.084 |
| K | lys | L-lysine | 0.064 |
| M | met | L-methionine | 0.029 |
| F | phe | L-phenylalanine | 0.035 |
| P | pro | L-proline | 0.041 |
| S | ser | L-serine | 0.040 |
| T | thr | L-threonine | 0.047 |
| W | trp | L-tryptophan | 0.011 |
| Y | tyr | L-tyrosine | 0.026 |
| V | val | L-valine | 0.079 |

**Table S1.** Amino acid frequencies

| name | millimoles / g DW |
|---|---|
| L-aspartate | 0.23 |
| L-asparagine | 0.23 |
| L-threonine | 0.24 |
| L-isoleucine | 0.27 |
| L-methionine | 0.14 |
| L-lysine | 0.32 |
| Total | 1.43 |

**Table S2.** Overall contents of aminoacids of the OAA family.

Since the consumption of a biomass component for growth is the content times the growth rate, we can estimate the demand for oxaloacetate for amino acid synthesis to be $1.43\,\mu$ millimoles per unit time and g of dry weight.

The concentration of ectoine in the cell is determined by the equation:

$$\frac{d[\text{ectoine}]}{dt} = V_{synt} - \mu\,[\text{ectoine}] \tag{S1}$$

where the negative term is the dilution that results from the increase of intracellular volume. Thus, cells in exponential growth reach a quasi-steady-state where the synthesis of ectoine is:

$$V_{synt} = \mu\,[\text{ectoine}] \tag{S2}$$

So the metabolic demand for oxaloacetate can be estimated from the growth rate and ectoine concentration.

The overview presented in figure 2 of the main text uses data from the literature (Dötsch et al., 2008), where both ectoine content and growth rate are given as a function of the salt concentration of the medium.

The oxaloacetate demands presented in figure 11 of the main text for the wild type, *H. elongata*-OAD and *H. elongata*-PPC use ectoine concentrations and growth rates measured in this study.

## 2 BIOMASS QUANTIFICATION

Simultaneous Thermal Analysis (STA) can be used to determine the biomass of small samples thanks to the use of a high precision balance. The composition of the materials leaving the sample as gas can be followed through spectrometry and the heat flow is measured to estimate the enthalpy of the processes taking place. This method allows to classify the loss of mass in each sample into water, biomass and ash content.

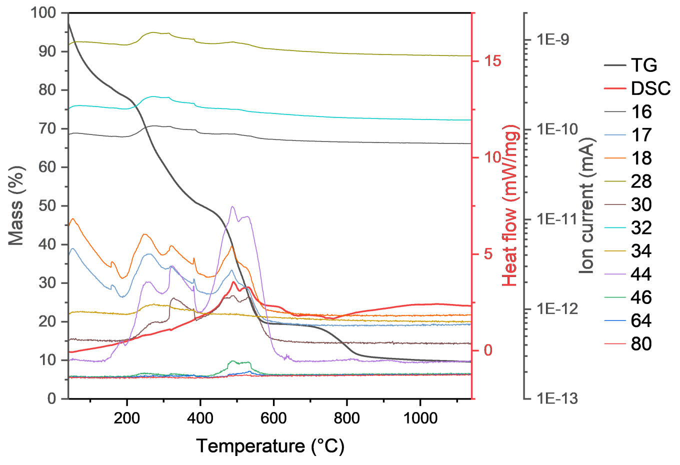


**Figure S1.** Simultaneous thermal analysis of dry biomass from *H. elongata* (OD 1.2). The mass is detected with a balance over a temperature range from 40 - 1140 °C with a heating rate of 1 K/min under air atmosphere. The heat uptake is compared to an empty crucible and the heatflow is plotted over the whole temperature range. Furthermore, different mass traces are recorded for the decomposition process with a quadrupole mass spectrometer which is coupled to the STA.

Through STA measurements, a correlation between OD600 and ash-free dry weight has been established, which has been used throughout this work to quantify biomass.

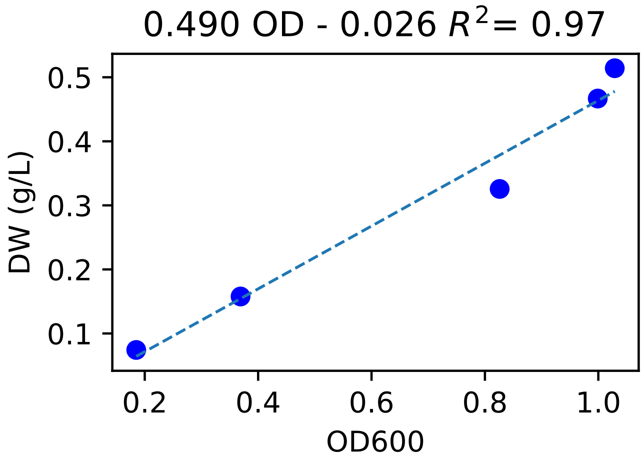


**Figure S2.** OD600 correlates with the ash free dry weight of *H. elongata*.

# 3 MODELING

## 3.1 Theory

The dynamics of metabolites in the cell can be represented by the equation:

$$\dot{\mathbf{x}} = \mathbf{S}\mathbf{v} \tag{S3}$$

where $\dot{\mathbf{x}}$ is a vector of metabolite concentrations, $\mathbf{v}$ a vector of reaction rates and $\mathbf{S}$ is a matrix of stoichiometric coefficients. For a culture growing in exponential phase, a quasi-steady-state is reached where the concentrations of all intermediary metabolites is constant.

$$\mathbf{S}\mathbf{v} = \mathbf{0} \tag{S4}$$

The technique known as Flux Balance Analysis (FBA) calculates flux distributions that optimize some physiologically meaningful goal – e.g. growth yield. Typically FBA solves the following linear program:

$$\begin{aligned} \max \quad & \\ & \mathbf{c}\,\mathbf{v} \\ \text{subject to:} \quad & \\ & \mathbf{S}\mathbf{v} = \mathbf{0} \\ & \boldsymbol{\alpha} \leq \mathbf{v} \leq \boldsymbol{\beta} \end{aligned} \tag{S5}$$

where $\mathbf{c}$ is a vector of constants and $\boldsymbol{\alpha}, \boldsymbol{\beta}$ are the lower and upper bounds for the reaction rates respectively.

Useful as it is, the application of this technique to *H. elongata* leaves many questions open regarding which of the predicted flux distributions are thermodynamically feasible and how costly it is to maintain the predicted fluxes (Kindzierski et al., 2017). This limitations can be overcome by extending equations S5 with thermodynamic constraints:

$$\mathbf{g} = \mathbf{g_0} + RT\,\ln\mathbf{a} \tag{S6}$$

$$\text{sign}(v_i)\,g_i < \text{MDF}\,\forall\,i \tag{S7}$$

Equation S6 defines a vector of molar free energies of the reactions ($\Delta G$) with $\mathbf{g_0}$ being the vector of standard free energies, $R$ is the gas constant and $T$ the temperature. vector **a** collects the activities of the metabolites. Activities are related to concentrations by the activity coefficients $a_i = \gamma_i\,x_i$, as explained in the main text. The activity of water is 1 in its pure state and decreases with ionic strength. This change is negligible within the scope of this study. Equation S7 ensures that the free energies of reactions are all negative in the direction in which they proceed. MDF is an acronym for Minimum Driving Force and it is a positive number with units of free energy that establishes the smallest acceptable magnitude for the free energies of reaction in the system. Setting MDF $= 0$ would ensure that all solutions of the problem are thermodynamically feasible, but imposing a minimum magnitude enables to filter solutions by cost. All other conditions being equal, reactions operating close to equilibrium require higher amounts of enzyme to maintain the same flux (Noor et al., 2014; Beard and Qian, 2007). This can easily be seen by writing the reaction rates in the following form:

$$v = (1-\theta)\,v_{max}\,\sigma(\mathbf{x}) \tag{S8}$$

All the traditional rate laws in enzyme kinetics can be factored in these terms (Noor et al., 2013, 2014) where $0 \leq \sigma(\mathbf{x}) \leq 1$ is a saturation function of arbitrary complexity, $v_{max}$ increases linearly with the amount of enzyme and $\theta = \exp(\Delta G/RT)$ is a measure of proximity to equilibrium. As a reaction approaches equilibrium, $\theta \to 1$, so higher amounts of enzyme will be needed to keep the same flux. The term $(1-\theta)$ can then be seen as a thermodynamic efficiency, for $\Delta G > RT$, the free energy has a minor impact on the efficiency of the enzyme but as soon as the free energy approaches $RT$, the efficiency decreases very fast. In this work we will designate the values of MDF using values such as $\Delta G_5$, $\Delta G_{10}$ and $\Delta G_{95}$ meaning the free energies associated to efficiencies of 5%, 10% or 95% respectively (Sehr et al., 2015). In other words, a flux distribution obtained for MDF $= \Delta G_{99}$ will have all its enzymes operating at a thermodynamic efficiency of at least 99% while in a flux distribution obtained for MDF $= \Delta G_{01}$, one or more enzymes can be operating only at 1% even at full saturation.

All the simulations in this study where conducted with different limits for the MDF ranging from $\Delta G_{01}$ to $\Delta G_{99}$.

## 3.2 The model

The model contains the reactions in table 3.2 identified by their KEGG codes (`www.genome.jp/kegg`) when available. Thermodynamic data for the reactants were obtained from equilibrator `http://equilibrator.weizmann.ac.il/` based on the dataset in (Alberty, 2003). Physiologically realistic range for the metabolite concentrations and cofactor ratios set as reported in (Vojinović and von Stockar, 2009; Noor et al., 2014). Most metabolites have concentrations constrained in the range 10 $\mu$M – 5 mM. Exception to these limits are: the proton concentrations, determined by the pH, water activity, which is kept to 1 in all cases, external sodium concentration, determined by the medium composition and

| Name | Equation |
|---|---|
| rn:R00200 | ATP + Pyruvate $\leftarrow$ PEP + ADP |
| rn:R00199 | Pyruvate + H2O + ATP $\rightarrow$ PEP + Phosphate + AMP |
| rn:R00342 | Malate + NAD $\leftrightharpoons$ H + NADH + Oxaloacetate |
| rn:R02164 | Q + Succinate $\leftrightharpoons$ QH2 + Fumarate |
| rn:R00341 | ATP + Oxaloacetate + H2O $\rightarrow$ ADP + $CO_2$ + PEP |
| rn:R08549 | NAD + 2Oxoglutarate + CoA + H2O $\rightarrow$ $CO_2$ + SuccinylCoA + H + NADH |
| rn:R00472 | CoA + Malate $\leftarrow$ H2O + Glyoxylate + AcetylCoA |
| rn:R00345 | Phosphate + Oxaloacetate $\leftrightharpoons$ $CO_2$ + PEP |
| rn:R00479 | Isocitrate $\leftrightharpoons$ Succinate + Glyoxylate |
| rn:R00209 | NAD + Pyruvate + CoA + H2O $\leftrightharpoons$ $CO_2$ + H + NADH + AcetylCoA |
| rn:R00405 | CoA + ATP + Succinate $\leftrightharpoons$ ADP + SuccinylCoA + Phosphate |
| rn:R01325 | Citrate $\leftrightharpoons$ H2O + cisAconitate |
| rn:R00267 | NADP + H2O + Isocitrate $\leftrightharpoons$ $CO_2$ + H + 2Oxoglutarate + NADPH |
| rn:R00351 | CoA + Citrate $\leftarrow$ H2O + AcetylCoA + Oxaloacetate |
| rn:R01900 | Isocitrate $\leftrightharpoons$ H2O + cisAconitate |
| rn:R01082 | Malate $\leftrightharpoons$ H2O + Fumarate |
| rn:R00214 | NAD + Malate + H2O $\leftrightharpoons$ NADH + H + Pyruvate + $CO_2$ |
| rn:R00216 | Malate + NADP + H2O $\leftrightharpoons$ $CO_2$ + H + Pyruvate + NADPH |
| ATP$d$ | ATP + H2O $\rightarrow$ ADP + Phosphate |
| rn:R00127 | ATP + AMP $\leftrightharpoons$ 2.0 ADP |
| NADPH$d$ | NADPH $\rightarrow$ NADP + H |
| NADH$d$ | NADH $\rightarrow$ NAD + H |
| ATPase | ADP + Phosphate + 4.0 $H_{ext}$ + H $\rightarrow$ 4.0 H + ATP + H2O |
| C1* | NADH + Q + 5.0 H $\rightarrow$ NAD + QH2 + 4.0 $H_{ext}$ |
| C3-4 | QH2 + 0.5 O2 + 6.0 H $\rightarrow$ Q + 6.0 $H_{ext}$ + H2O |
| rn:R00235 | ATP + Acetate + CoA $\leftrightharpoons$ AMP + PPi + AcetylCoA |
| rn:R00217 | 2.0 Na + Oxaloacetate + H2O + $H_{ext}$ $\leftrightharpoons$ $CO_2$ + Pyruvate + 2.0 $Na_{ext}$ + H |
| Napump32 | 3.0 $H_{ext}$ + 2.0 Na $\leftrightharpoons$ 3.0 H + 2.0 $Na_{ext}$ |
| Napump21 | 2.0 $H_{ext}$ + Na $\leftrightharpoons$ 2.0 H + $Na_{ext}$ |
| Napump11 | $H_{ext}$ + Na $\leftrightharpoons$ H + $Na_{ext}$ |
| Na-Nqr | NADH + 2.0 Na + Q + 3.0 H $\rightarrow$ NAD + 2.0 $Na_{ext}$ + QH2 + 2.0 $H_{ext}$ |

**Table S3.** List of reactions in the model. * only present in the model without sodium.

| Metabolite | Lower bound (mM) | Upper bound (mM) |
|---|---|---|
| Phosphate | 7.5 | 8.5 |
| $CO_2$ | 0.001 | 0.03 |
| $Na_{cyt}$ | 10 | 20 |
| O2 | 0.01 | 0.3 |
| Q | 0.0001 | 20 |
| $QH_2$ | 0.0001 | 20 |

**Table S4.** All the oither metabolites are constrained to the range 10 $\mu$M – 5 mM. The bounds for quinones in reduced and oxidized forms were left extremely wide to guarantee the realistic operation of the electron transport chain.

those summarized in table 3.2. The membrane potential is assumed to be 150 mV Jol et al. (2010); Henry et al. (2007).

In addition to the constraints on metabolite concentrations, the ratios of cofactors were constrained as follows: ATP/ADP = 10, ADP = AMP, NADPH/NADP = 10 and NADH/NAD = 0.1 (Vojinović and von Stockar, 2009; Noor et al., 2014)

### 3.3 A model without sodium

As a baseline to understand the role of sodium, a model was formulated lacking all the reactions in which sodium participates. This includes Oad, all the sodium/proton antiporters and the sodium dependent Na-Nqr. In order to have a functional electron transport chain, the usual reaction catalyzed by complex I was added to replace Na-Nqr.

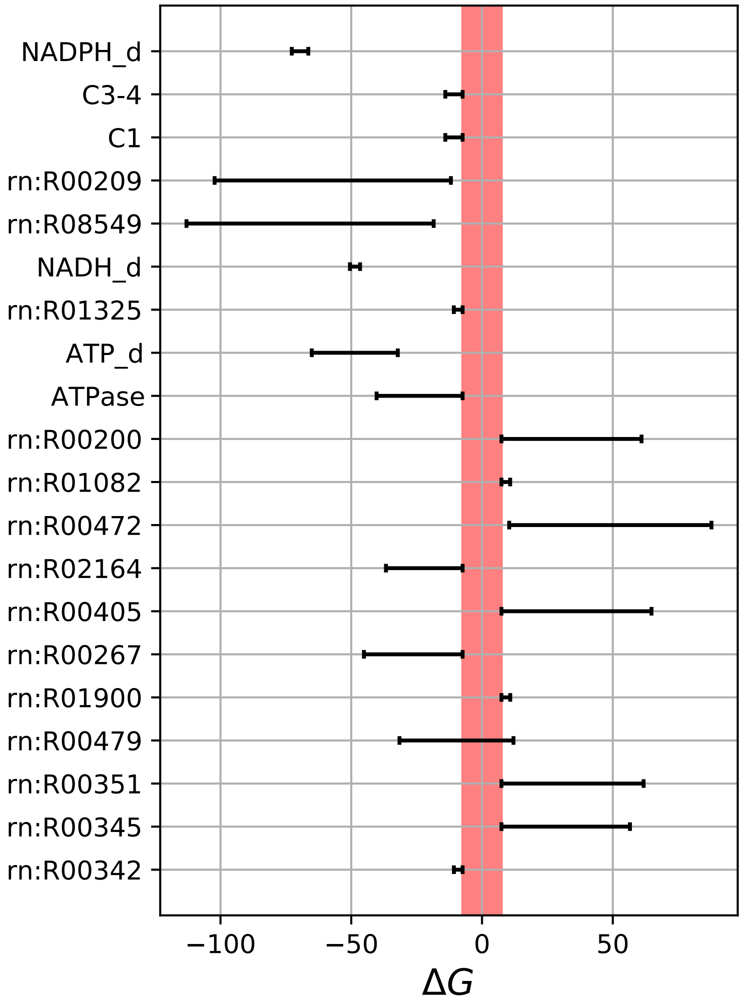


**Figure S3.** Free energy ranges for all solutions of the model without sodium dependent processes with the condition MDF $= \Delta G_{95}$. Only reactions that are active in at least one solution shown. Shaded area represents the interval between -MDF and +MDF.

The set of solutions for this model can be divided in two groups represented in figures 5 and 6 in the main text. In one group, the anaplerotic reaction Ppc (rn:R00345) is active and in the other group, the corresponding flux is carried by the glyoxilate shunt (rn:R00472 + rn:R00479). Figure S3 shows the intervals of admissible $\Delta G$ values for all these solutions. The intervals are limited by the MDF, with the intervals for reactions that remain positive in all solutions being on the negative side and reactions that remain negative having their intervals$\Delta G$ on the positive side. Only one of the enzymes in the glyoxilate cycle (rn:R00479) has an interval that spans both sides, this is a consequence mathematical formulation of the problem. When a reaction is not active in a solution, its $\Delta G$ is not constrained. Thus, for those solutions

in which this reactions carries flux, its free energy is constrained to the subset left of the -MDF limit. The free energy ranges are also helpful to recognize the thermodynamic bottlenecks of the network. As the network is forced to operate at higher levels of thermodynamic efficiency, the admissible intervals for $\Delta G$ narrow down much faster for some enzymes as they are brought to their thermodynamic limits of operation. In this case we can see several such reactions, including but not limited to Mdh, an enzyme that has been described as one of the most difficult steps in the central metabolism.

### 3.4 The full model

The simulations were repeated after re-establishing the sodium dependent processes and removing the sodium independent complex I. Simuations were conducted at the four salt concentrations that were studied experimentally: 0.17 M, 0.5 M, 1 M and 2 M. For extracellular sodium concentrations of 0.5 M and above, Oad can carry the anaplerotic flux enabling higher oxaloacetate yields (see figure 7 in the main text).

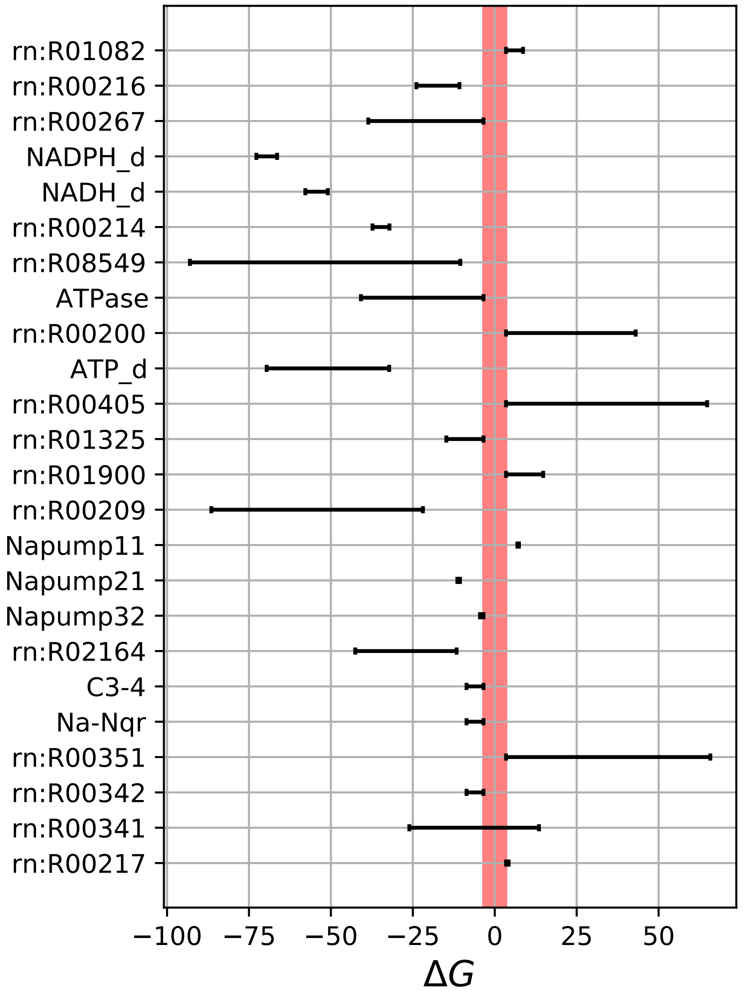


**Figure S4.** Free energy ranges for all solutions of the full model at 1 M NaCl with the condition MDF = $\Delta G_{75}$. Only reactions that are active in at least one solution shown. Shaded area represents the interval between -MDF and +MDF.

The free energy intervals associated to this solution, shown in figure S4 for 1 M NaCl and MDF = $\Delta G_{75}$ clearly indicate that Oad (rn:R00217) is one of the main thermodynamic bottlenecks of the network. If we

search for solutions able to operate at higher thermodynamic efficiency, Oad is no longer viable and the model offers lower yield alternatives such as the flux distribution shown in figure S5 for MDF $= \Delta G_{90}$.

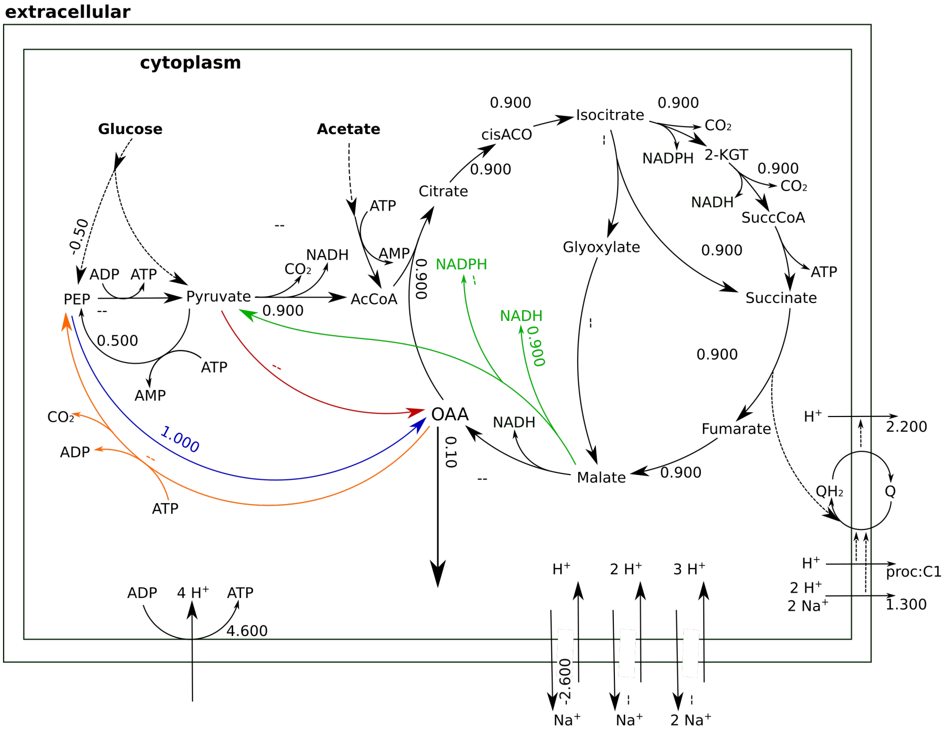


**Figure S5.** Flux distributions for optimal OAA yield at 1 M NaCl imposing the condition MDF $= \Delta G_{90}$. All fluxes are normalized per unit of glucose input.

The free energies associated to this solution show that in spite of the more stringent thermodynamic constraints ($\Delta G_{90}$ vs $\Delta G_{75}$) the intervals in this case are wider.

Finally, the operation of Oad is completely infeasible in all simulations for salt concentrations of 0.17 M. Figure S7 shows a flux distribution obtained for $\Delta G_5$ where the anaplerotic flux goes through Ppc instead of Oad.

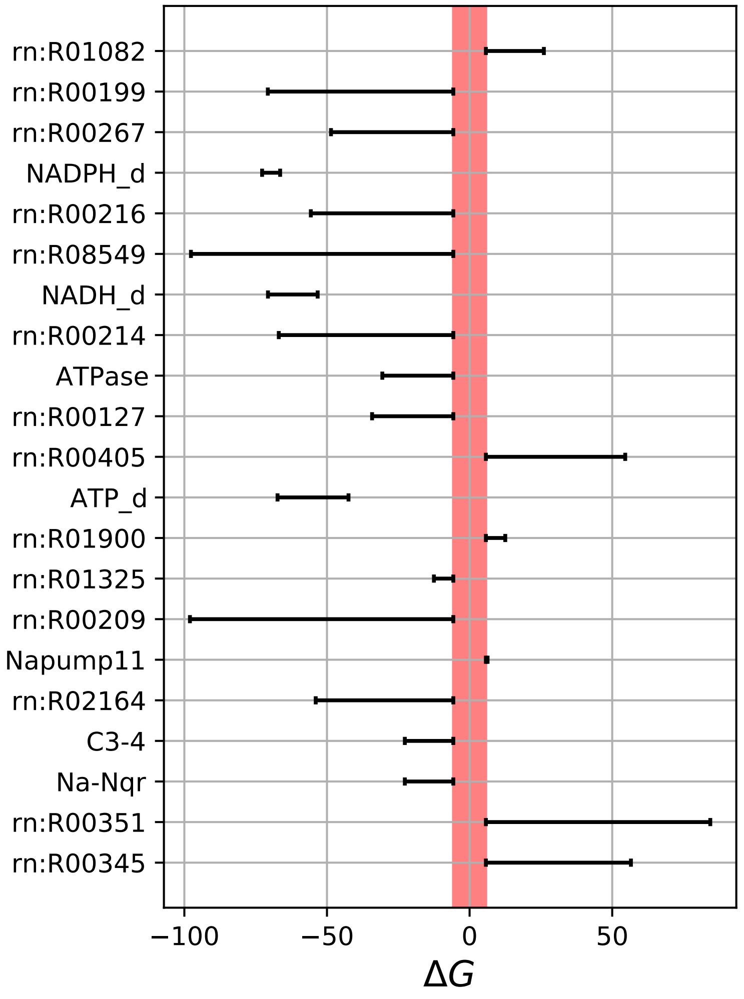


**Figure S6.** Free energy ranges for all solutions of the full model at 1 M NaCl with the condition MDF = $\Delta G_{90}$. Only reactions that are active in at least one solution shown. Shaded area represents the interval between -MDF and +MDF.

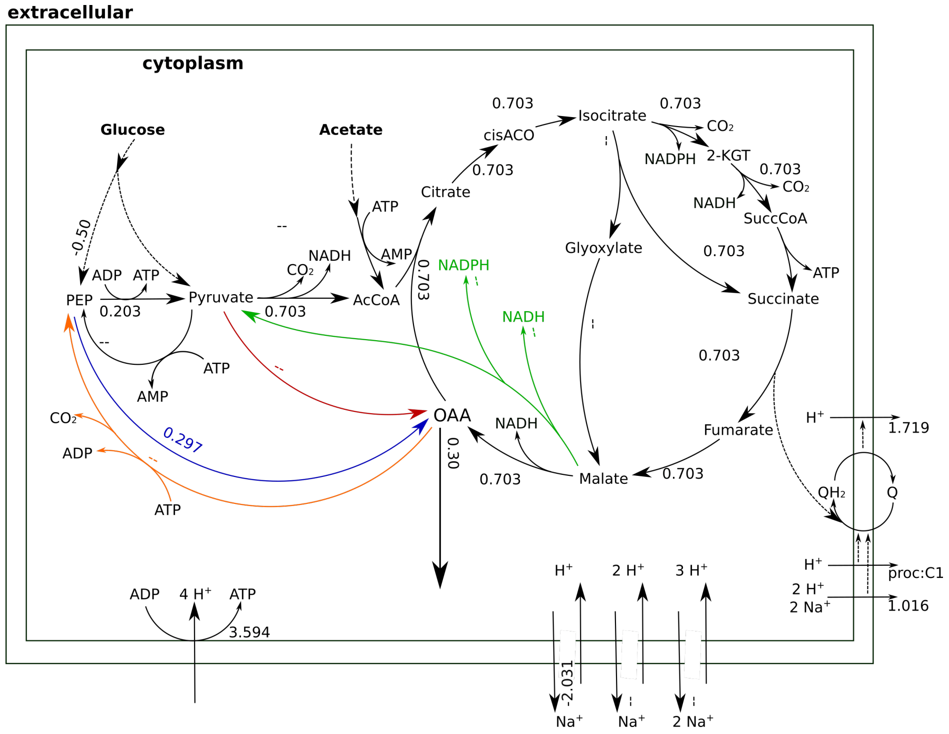


**Figure S7.** Flux distributions for optimal OAA yield at 0.17 M NaCl imposing the condition MDF $= \Delta G_5$. All fluxes are normalized per unit of glucose input.

# 4 GROWTH CURVES

## 4.1 Experiments in microtiter plate

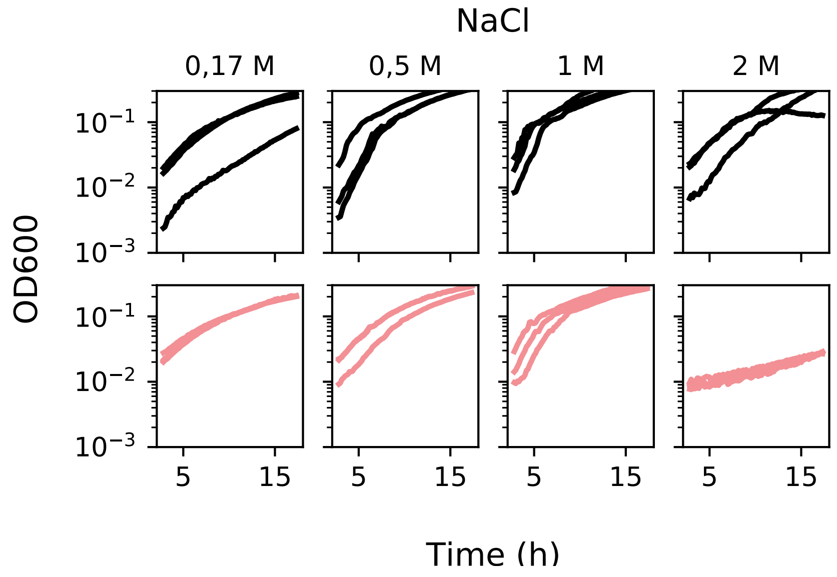


**Figure S8.** *H. elongata*-OAD on glucose

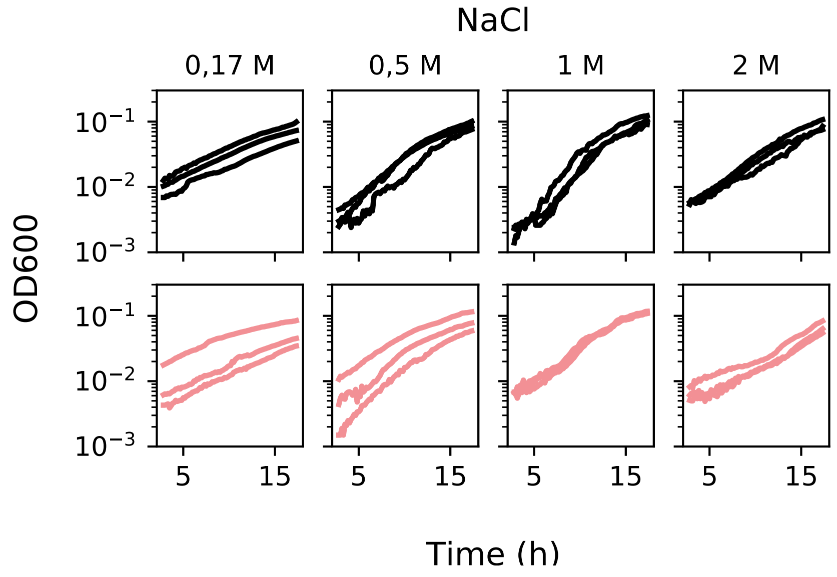


**Figure S9.** *H. elongata*-OAD on acetate

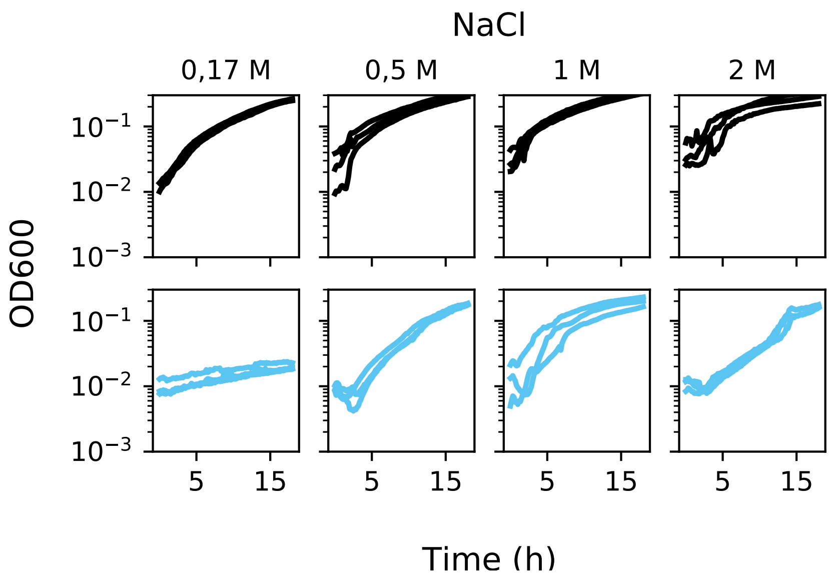


**Figure S10.** *H. elongata*-PPC on glucose

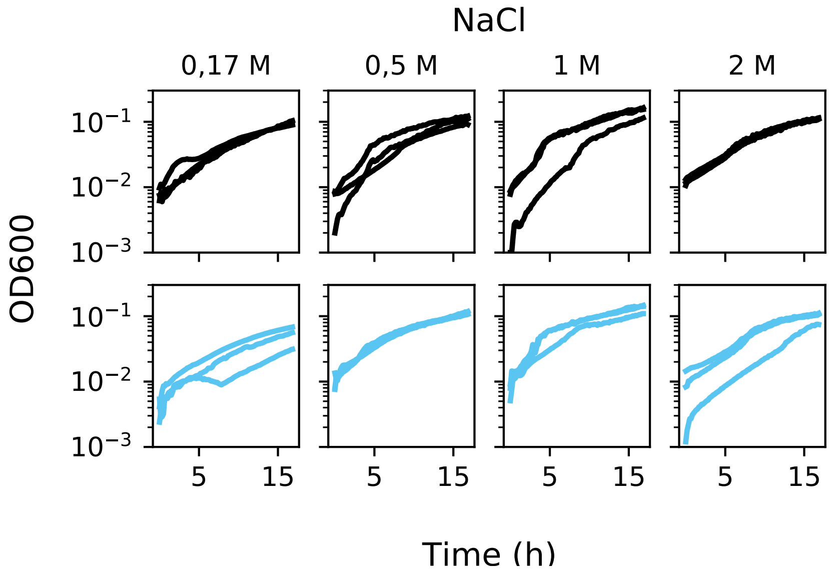


**Figure S11.** *H. elongata*-PPC on acetate

### 4.2 Additional experiments in microtiter plate

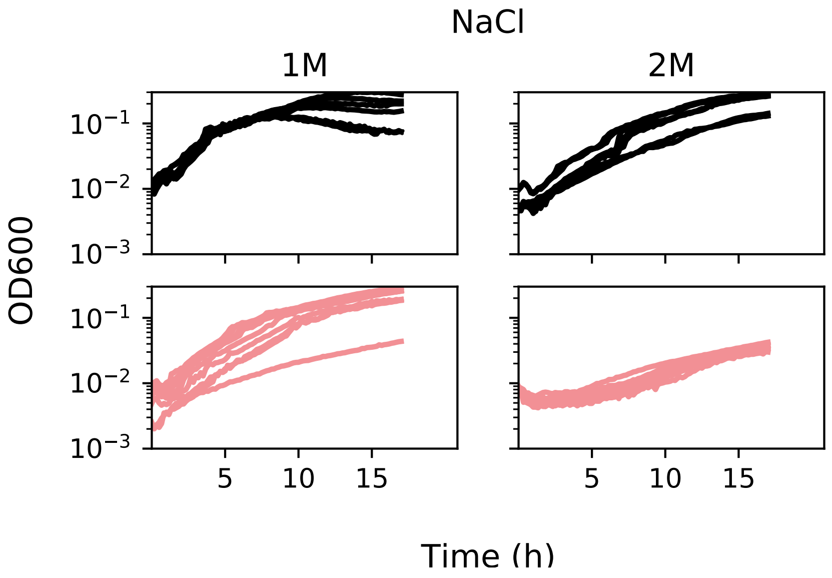


**Figure S12.** *H. elongata*-OAD on glucose

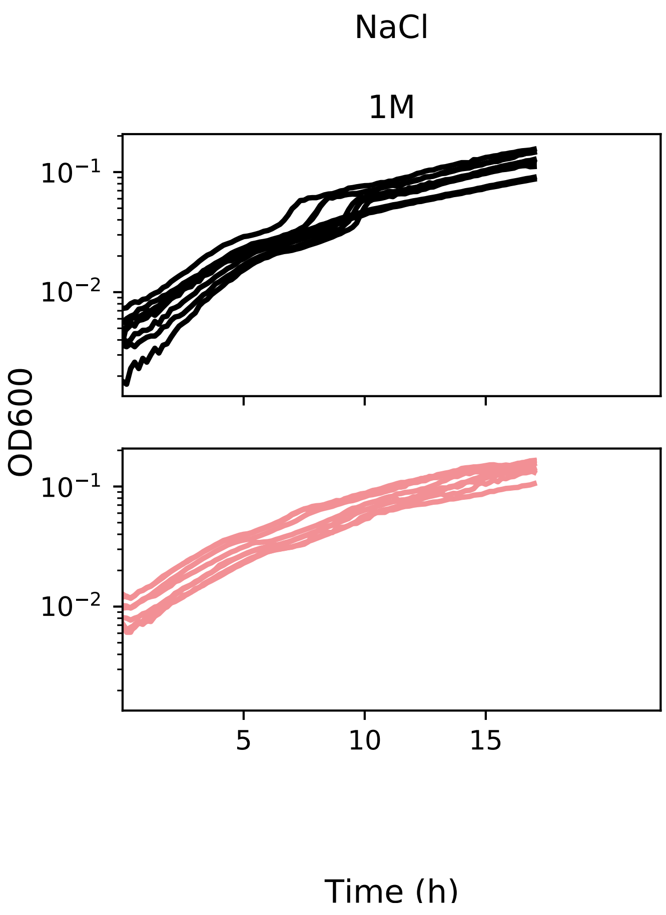


**Figure S13.** *H. elongata*-OAD on acetate

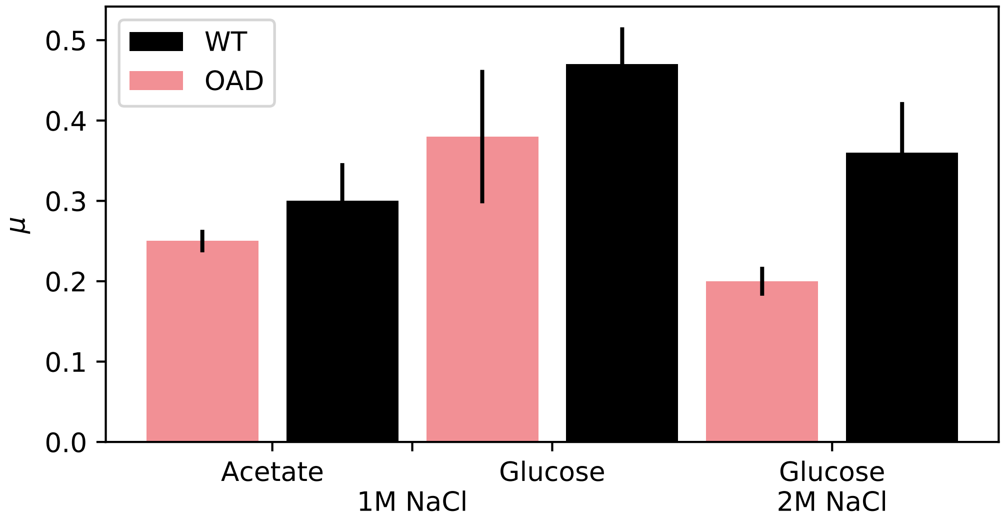


**Figure S14.** Summary of maximum growth rates for *H. elongata*-OAD and WT in the additional experiments

### 4.3 Experiments in flask

| csource | strain | salt | growth | err |
|---|---|---|---|---|
| OAD | Ac | 2 M | 0.12 | 0.003 |
| | Glc | 2 M | 0.09 | 0.004 |
| PPC | Ac | 0.17 M | 0.16 | 0.018 |
| | Glc | 0.17 M | 0.06 | 0.037 |
| | | 2 M | 0.13 | 0.014 |
| WT | Ac | 0.17 M | 0.19 | 0.004 |
| | Glc | 0.17 M | 0.28 | 0.007 |
| | | 2 M | 0.25 | 0.011 |

**Table S5.** Summary of maximum growth rates in flask experiments

#### 4.3.1 Growth at high salt (2 M NaCl)

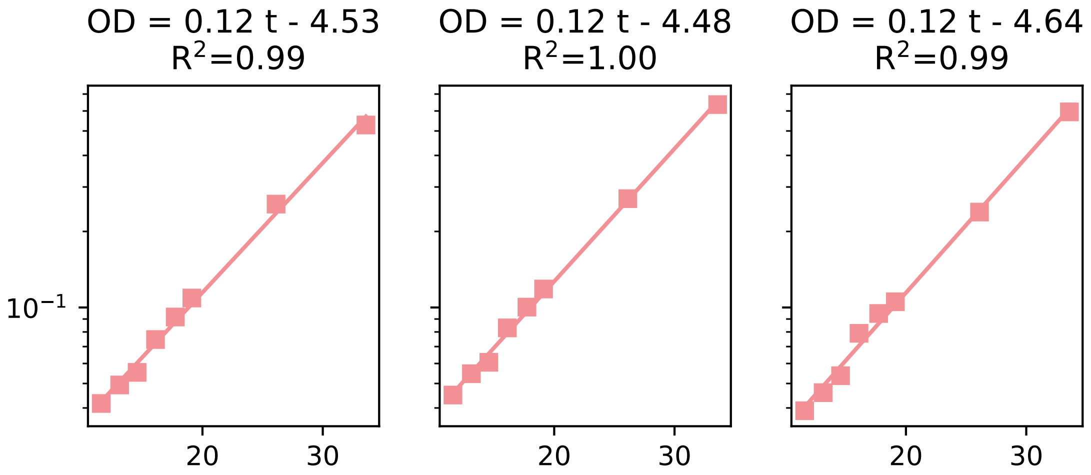


**Figure S15.** *H. elongata*-PPC on acetate

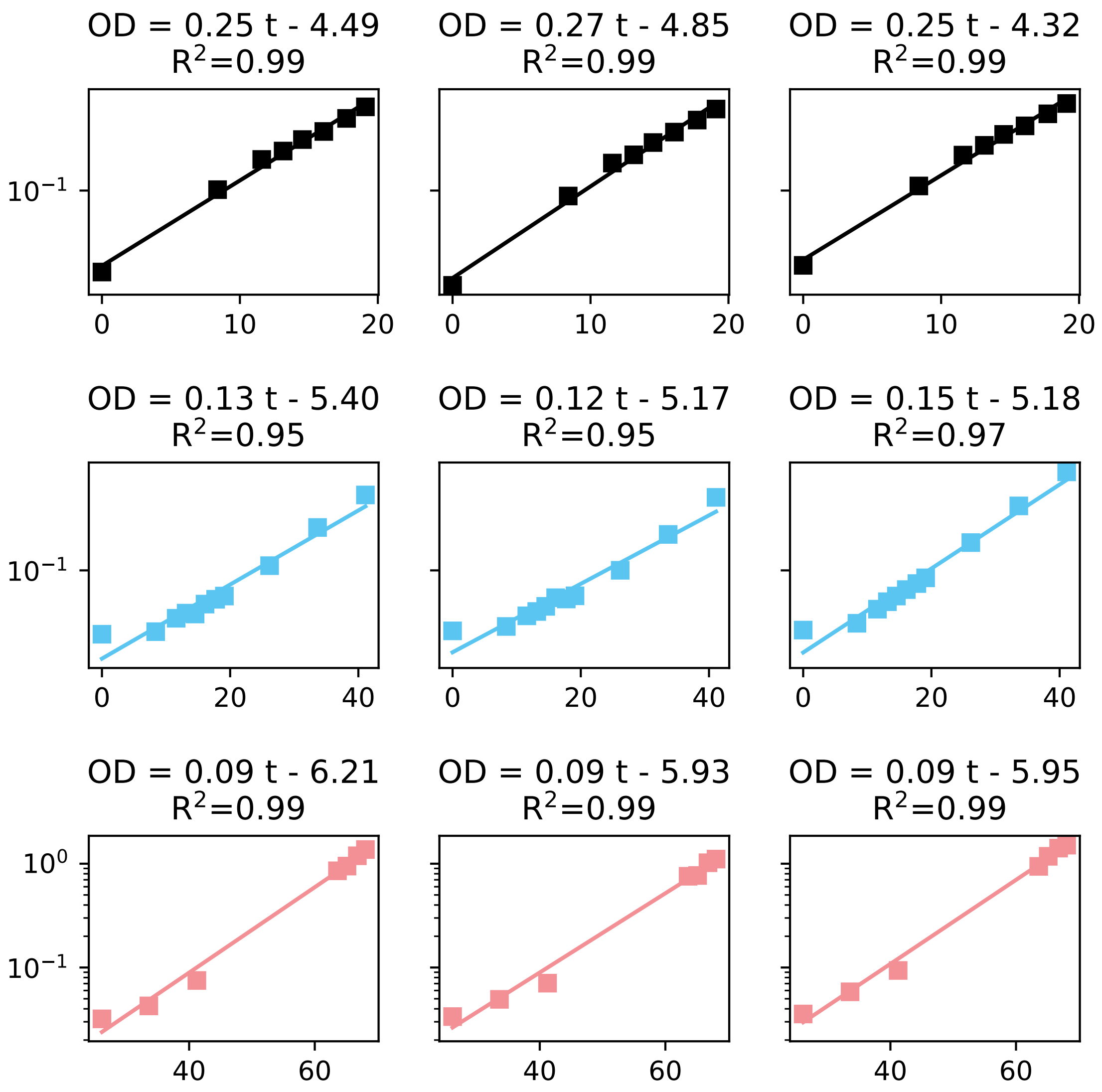


**Figure S16.** *H. elongata*-PPC, *H. elongata*-PPC and WT on glucose

#### 4.3.2 Growth at low salt (0.17 M NaCl)

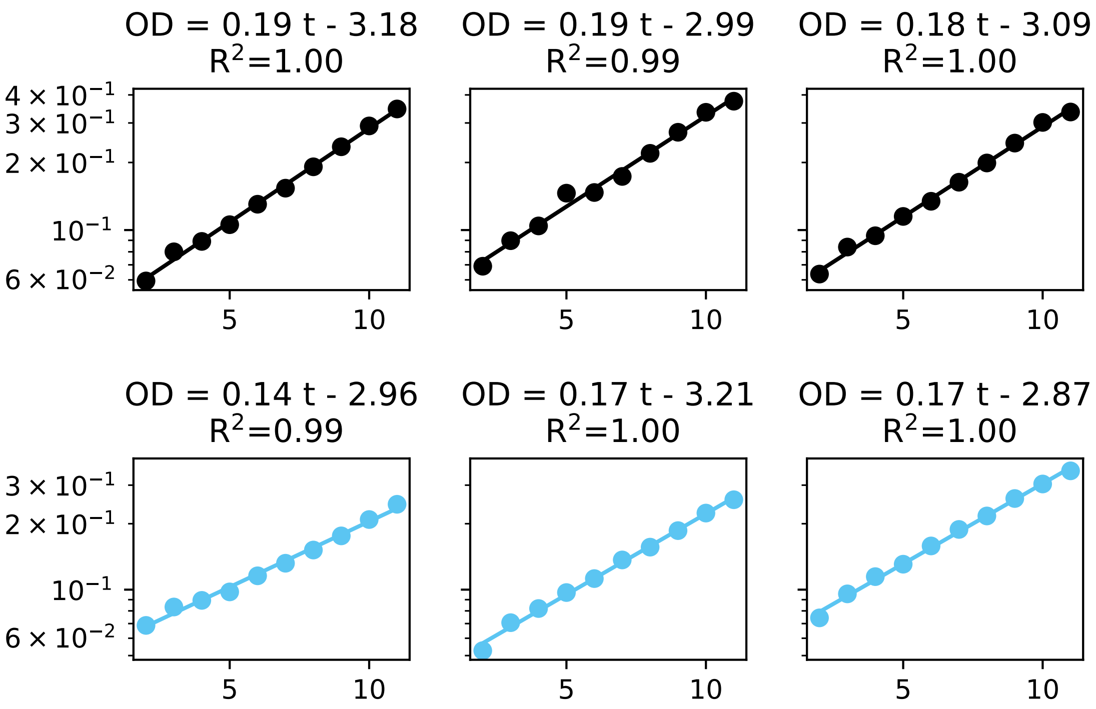


**Figure S17.** *H. elongata*-PPC on acetate

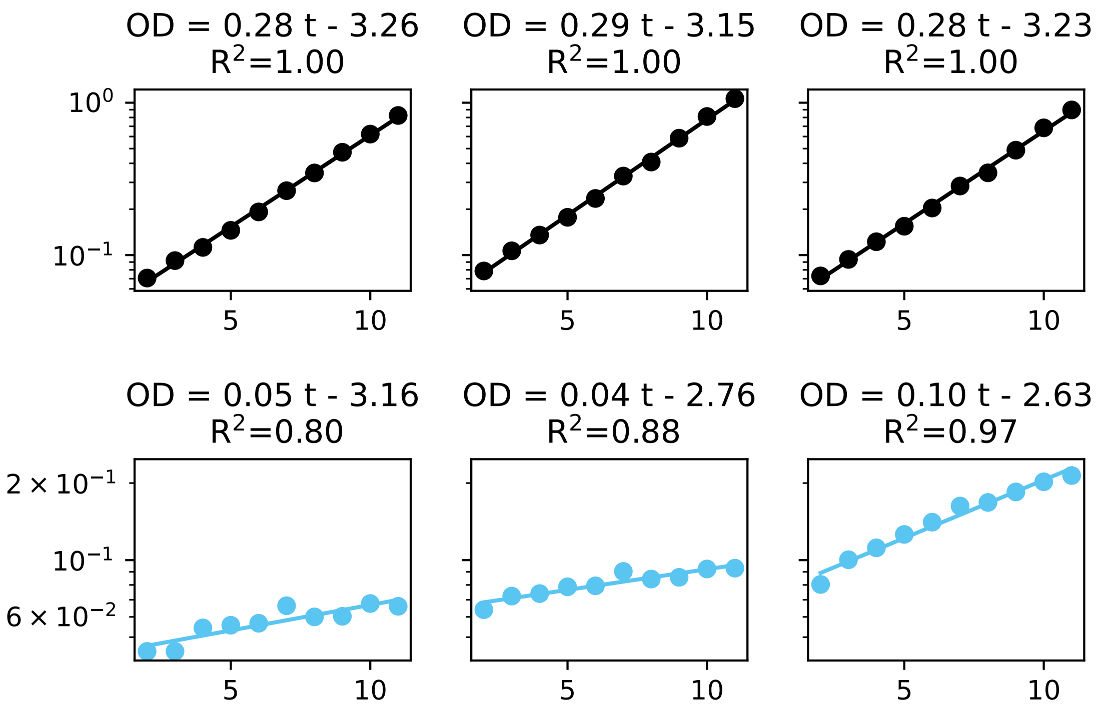


**Figure S18.** *H. elongata*-PPC on glucose

## REFERENCES


Alberty, R. A. (2003). *Thermodynamics of biochemical reactions* (John Wiley & Sons)

Beard, D. A. and Qian, H. (2007). Relationship between thermodynamic driving force and one-way fluxes in reversible processes. *PloS one* 2, e144

Dötsch, A., Severin, J., Alt, W., Galinski, E. A., and Kreft, J.-U. (2008). A mathematical model for growth and osmoregulation in halophilic bacteria. *Microbiology* 154, 2956–2969

Henry, C. S., Broadbelt, L. J., and Hatzimanikatis, V. (2007). Thermodynamics-based metabolic flux analysis. *Biophysical journal* 92, 1792–1805

Jol, S. J., Kümmel, A., Hatzimanikatis, V., Beard, D. A., and Heinemann, M. (2010). Thermodynamic calculations for biochemical transport and reaction processes in metabolic networks. *Biophysical journal* 99, 3139–3144

Kindzierski, V., Raschke, S., Knabe, N., Siedler, F., Scheffer, B., Pflüger-Grau, K., et al. (2017). Osmoregulation in the halophilic bacterium *Halomonas elongata*: A case study for integrative systems biology. *PLOS ONE* 12, e0168818

Neidhardt, F. C. and Umbarger, H. E. (1996). *Escherichia coli and Salmonella typhimurium; Cellular and Molecular Biology* (American Society for Microbiology Publishers), chap. Chemical Composition of *Escherichia coli*. 2 edn.

Noor, E., Bar-Even, A., Flamholz, A., Reznik, E., Liebermeister, W., and Milo, R. (2014). Pathway thermodynamics highlights kinetic obstacles in central metabolism. *PLoS computational biology* 10, e1003483

Noor, E., Flamholz, A., Liebermeister, W., Bar-Even, A., and Milo, R. (2013). A note on the kinetics of enzyme action: a decomposition that highlights thermodynamic effects. *FEBS letters* 587, 2772–2777

Sehr, C., Kremling, A., and Marin-Sanguino, A. (2015). Design principles as a guide for constraint based and dynamic modeling: Towards an integrative workflow. *Metabolites* 5, 601–635

Vojinović, V. and von Stockar, U. (2009). Influence of uncertainties in ph, pmg, activity coefficients, metabolite concentrations, and other factors on the analysis of the thermodynamic feasibility of metabolic pathways. *Biotechnology and bioengineering* 103, 780–795
